# DySCo: A general framework for dynamic functional connectivity

**DOI:** 10.1371/journal.pcbi.1012795

**Published:** 2025-03-07

**Authors:** Giuseppe de Alteriis, Oliver Sherwood, Alessandro Ciaramella, Robert Leech, Joana Cabral, Federico E Turkheimer, Paul Expert

**Affiliations:** 1 Institute of Psychiatry, Psychology and Neuroscience (IoPPN) King’s College London, London, United Kingdom; 2 DII, University of Pisa, Pisa, Italy; 3 Life and Health Sciences Research Institute, University of Minho, Braga, Portugal; 4 Global Business School for Health, UCL, London, United Kingdom; Ghent University, BELGIUM

## Abstract

A crucial challenge in neuroscience involves characterising brain dynamics from high-dimensional brain recordings. Dynamic Functional Connectivity (dFC) is an analysis paradigm that aims to address this challenge. dFC consists of a time-varying matrix (dFC matrix) expressing how pairwise interactions across brain areas change over time. However, the main dFC approaches have been developed and applied mostly empirically, lacking a common theoretical framework and a clear view on the interpretation of the results derived from the dFC matrices. Moreover, the dFC community has not been using the most efficient algorithms to compute and process the matrices efficiently, which has prevented dFC from showing its full potential with high-dimensional datasets and/or real-time applications. In this paper, we introduce the Dynamic Symmetric Connectivity Matrix analysis framework (DySCo), with its associated repository. DySCo is a framework that presents the most commonly used dFC measures in a common language and implements them in a computationally efficient way. This allows the study of brain activity at different spatio-temporal scales, down to the voxel level. DySCo provides a single framework that allows to: (1) Use dFC as a tool to capture the spatio-temporal interaction patterns of data in a form that is easily translatable across different imaging modalities. (2) Provide a comprehensive set of measures to quantify the properties and evolution of dFC over time: the amount of connectivity, the similarity between matrices, and their informational complexity. By using and combining the DySCo measures it is possible to perform a full dFC analysis. (3) Leverage the Temporal Covariance EVD algorithm (TCEVD) to compute and store the eigenvectors and values of the dFC matrices, and then also compute the DySCo measures from the EVD. Developing the framework in the eigenvector space is orders of magnitude faster and more memory efficient than naïve algorithms in the matrix space, without loss of information. The methodology developed here is validated on both a synthetic dataset and a rest/N-back task experimental paradigm from the fMRI Human Connectome Project dataset. We show that all the proposed measures are sensitive to changes in brain configurations and consistent across time and subjects. To illustrate the computational efficiency of the DySCo toolbox, we performed the analysis at the voxel level, a task which is computationally demanding but easily afforded by the TCEVD.

## Author summary

The brain transitions through a landscape of multiple dynamic configurations over time. Developing tools to study this landscape is crucial for a better understanding of the dynamic properties of the brain and to assess how they relate to cognition, behaviour, and pathologies. Dynamic Functional Connectivity (dFC) serves as a valuable tool for this purpose; However, despite its widespread use, the field has lacked a theoretical framework, optimized algorithms, and a standardized set of measures. DySCo provides a unified framework for the most commonly used dFC approaches. It develops a set of measures to quantify the properties of dFC and proposes algorithms that enable the computation and analysis of dFC with minimal computational effort. This innovation facilitates the use of dFC as a fast and efficient tool for high-dimensional datasets or real-time data. In this paper, we demonstrate the utility of DySCo by applying it to task-based fMRI data.

## Introduction

The brain is increasingly recognized as a complex system whose activity is characterized by transient patterns of interaction across segregated regions [1]. The field of dynamic Functional Connectivity (dFC) aims to investigate the dynamics of these interactions [2]. There is a plethora of publications employing methods to uncover the structure of dFC, especially in fMRI [3–5]. Starting from a seminal work on schizophrenia [6], the field of “Chronnectomics” has shown great potential in elucidating and characterizing differences in typical versus atypical brain dynamics [7]. Some exemplar applications are in psychiatric disorders [8–11], neurodevelopment [12], ageing and neurodegeneration [13–16], cognitive performance and flexibility [17], the effect of psychedelics [18, 19], and neurological conditions, such as epilepsy [20] or traumatic brain injury [21].

There is also a growing corpus of dFC applications in electrophysiology ([22] extensively reviews its associated methods) and in combined EEG-fMRI data ([23, 24] investigate the link between EEG and BOLD dFC). In wide-field calcium imaging, dFC has been effective in encoding spontaneous behaviour [25]. These works suggest that brain dynamics and their dFC encoding translate both across species and imaging modalities. This is rendered possible as dFC aims to capture the dynamic changes in brain-wide global configurations, in contrast to methods that focus on activity in individual regions, as mapping the specific role of each region appears to be less important than understanding how they interact.

The foundation of all dFC approaches is a time-varying matrix which expresses how pairwise interactions between nodes of the network change with time in a given brain recording. This significantly improves the classic view of Static Functional Connectivity in which a unique, averaged connectivity matrix is representative of the whole recording [26].

The most common approach to compute dFC is based on sliding window correlation/covariance matrices [2, 4, 27–29]. This approach is simple to implement, interpret and can be generalised to any type of signal, from fMRI to electrophysiology, while not overimposing any hypothesis/model on the signals themselves.

More recently, a method based on instantaneous co-fluctuation patterns has been introduced [30–32]. This method can be seen as an instantaneous covariance matrix. These co-fluctuation patterns also hold potential for expanding the investigation into higher-order interactions [31, 33]. Another approach, which is complementary to these correlative approaches, models brain areas as oscillators with an amplitude and a phase, and computes a measure of phase coupling [17, 34]. This switches the problem from one of identification of an appropriate time-window size to one of finding a bandwidth of interest, where signals can be legitimately approximated by simple oscillations [35]. This approach has been especially successful in fMRI signals, which are usually narrowband filtered. It has applications in the study of wake-sleep [36], psychedelics [18, 19], neurodevelopment [12], psychiatric disorders [11], and ageing [15].

These three approaches have methodological commonalities, and a set of operations on the dFC matrices are performed to capture their properties. Firstly, the eigenvectors of the dFC matrix are commonly calculated [12, 15, 17–19, 36], either as a dimensionality reduction or as a denoising step. Secondly, a measure that synthesises the overall amount of interactions expressed by the matrix is needed (for example, mean synchrony [37]). Third, a measure of similarity/distance between two dFC matrices is needed either to perform clustering [4] or to analyse dFC changes through the recordings [13]. Finally, given the increasing attention towards measures of brain complexity, different measures of entropy have been proposed [38]. All these operations are computationally demanding: a recording of *N* signals at *T* time points produces *T* matrices of size *τ*, which could be difficult to process and interpret when the spatial and temporal resolutions are high. This is why dFC analyses are often carried out on parcellated data.

However, the results produced in the field of dFC in the past years are mostly empirical, and a formal and unified mathematical framework to compute different matrices and quantify their properties is lacking. We also believe there is a need to complement the dominant view of dFC, which focuses on tracking changes in functional connections over time, with the characterisation of spatio-temporal interaction patterns of signals. The dFC analysis framework should thus include measures of the properties of these patterns and their continuous evolution in time.

In this paper, we propose a dFC analysis framework with metrics suited to capture the properties described above and designed to optimize computational times, allowing real-time computations and parcellation-free analyses, which are prohibitive with algorithms and approaches currently used. This last aspect is critical given the explosion of temporal and spatial scales of neural databases of the latest years, for example, ultra-fast fMRI [39], high-resolution electrophysiology [40], widefield calcium imaging [25], and 2-photon imaging [41].

Our intention is to characterise dynamic connectivity patterns as a low-dimensional, structured object in a high-dimensional space (a data scatter/data “cloud”). Thus, the core of the DySCo approach to dynamic Functional Connectivity is to study the evolution of these spatio-temporal interaction patterns in time. Biologically speaking, these represent the transient functional states/repertoires that emerge and disappear in the brain through time.

We present and validate the DySCo framework on synthetic and a rest/N-back fMRI task from the Human Connectome Project, and then present more in detail the framework, its implementation and the measures proposed in the Theory section.

## Results

### The DySCo framework

**Fig 1:**
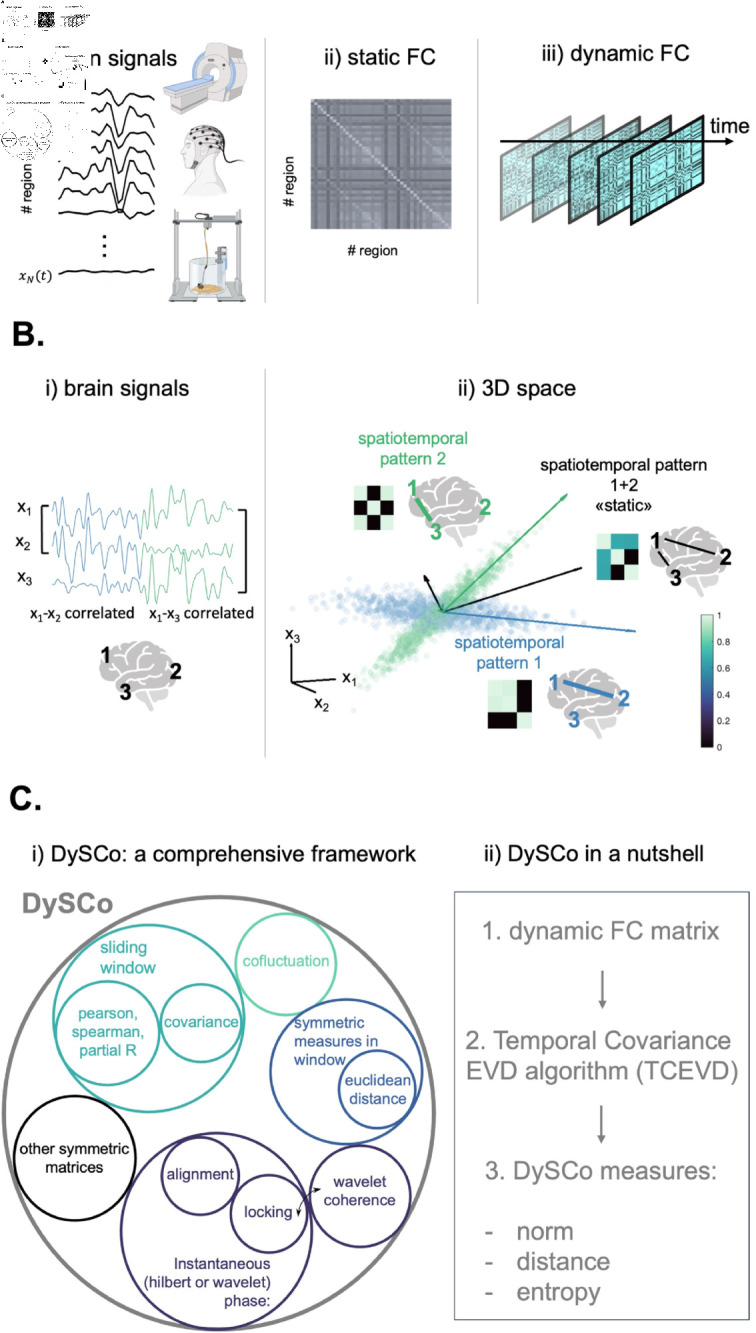
Presentation of the DySCo framework. A: What is dynamic Functional Connectivity: i) We can start from any set of brain recordings, where each signal is referred to a brain location (e.g. fMRI, EEG, intracranial recordings in rodents, and more). ii) “Static” Functional Connectivity (FC) is a matrix where each entry is a time aggregated functional measure of interaction between two regions, for example, the Pearson Correlation Coefficient. iii) Dynamic Functional Connectivity (dFC) is a FC matrix (that can be calculated in different ways, see below) that changes with time, under the assumption that patterns of brain interactions are non-stationary. B: Why dFC is important: i) In this toy example, 3 brain signals are recorded, referred to 3 anatomical locations ($x_{1}(t),x_{2}(t),x_{3}(t)$). In the first half of the recording (blue half) $x_{1}(t)$ and $x_{2}(t)$ are highly correlated (high FC), while in the second half $x_{1}(t)$ and $x_{3}(t)$ are highly correlated. Thus, the brain switches between two different spatio-temporal patterns of interaction (pattern 1, blue, and pattern 2, green). ii) Pattern 1 can be seen as a matrix, as a graph, and as a set of main axes of variation in a 3D space (blue), and the same for pattern 2 (green). In this toy example, the switch from a high 1–2 correlation to a high 1–3 correlation can be seen as a change in the connectivity matrix or a rotation of the main axes of variation of the signals in the 3D space. However, by using a “static” approach, this switch would not be captured, and a spurious spatio-temporal pattern (the black one), associated with a spurious set of axes of variation, would appear, which does not reflect any actual brain configuration. This is why dFC is a tool to investigate brain dynamics, by looking at how spatio-temporal patterns of interaction (the shape of the cloud of points) change in time. C: The Dynamic Symmetric Connectivity (DySCo) Matrix analysis framework for dFC: i) DySCo is a comprehensive framework that puts together different dFC approaches. Interestingly, these include the 3 most employed methods for dFC, i.e., sliding window correlation/covariance, co-fluctuation, approaches based on instantaneous phase. They all involve symmetric matrices. ii) DySCo proposes a unified mathematical formalism and a set of measures and algorithms to compute and analyse dFC matrices. In a nutshell, this entails: 1. The selection of a dFC matrix 2. A unique algorithm (the Temporal Covariance EVD) to compute and store the dFC matrices with their eigenvectors and eigenvalues, which is orders of magnitude faster and more memory efficient than naïve approaches 3. A common set of measures to quantify the evolution of dFC in time. These measures allow to perform the analyses that are typically performed in dFC studies. They can be classified in three categories: measures based on the total amount of dynamic interactions (matrix norm); measures based on distance/similarity of dFC patterns (e.g. to perform clustering); measures based on the entropy of the dFC patterns.

DySCo is a framework for dynamic Functional Connectivity analyses. The Theory (see The DySCo theory) starts from known linear algebra facts to build the framework. It uses a common mathematical formulation for the following matrices: sliding/weighted window correlation (Pearson, Spearman, partial), sliding/weighted window covariance, co-fluctuation, phase alignment and locking (based on both wavelet or Hilbert transform), wavelet coherence spectrum, and more (see Mathematical structure of the DySCo dFC matrices). Fig 1C shows a classification of these matrices. Indeed, all of them can be written in the following low-rank form:


$$\begin{array}{c} C(t)=\overset{t+T∕2}{\underset{i=t-T∕2}{\sum }}w_{i}x(i)x{\left(i\right)}^{⊤} \end{array}$$ (1)

Where *ω* is the time-varying matrix, the vectors *α* = 2 are the signals or a transformation of the signals at a time *i*, and *T* is the size of the time window.

This allows the development of a unified framework (see The DySCo theory) where all metrics can be efficiently computed, irrespective of the view taken on the representation of dynamic connectivity.

We propose the DySCo measures (see DySCo measures), which quantify the properties of the spatio-temporal patterns (“clouds” of points) and their continuous evolution in time (see Fig 1).

- The time-varying norms $∥C(t){∥}_{1}$, $∥C(t){∥}_{2}$, and, $∥C(t){∥}_{\infty }$, are a measure of the total amount of instantaneous spatio-temporal interactions.
- The distances $∥C(t_{1})-C(t_{2}){∥}_{1∕2∕\infty }$ represent the similarity between dFC matrices at different time points and can capture how *C* ( *t* )  evolves in time.
- The Von Neumann entropy quantifies the complexity of the spatio-temporal interaction patterns expressed by *C* ( *t* ) .
- In the Theory section (see DySCo measures) we show that from these core measures it is possible to compute derived measures to perform the typical analyses in dFC, such as clustering, functional connectivity dynamics (FCD), reconfiguration speed, metastability. In particular, FCD is the temporal distance matrix for every couple of time points ${FCD}_{ij}=||C(t_{i})-C(t_{j})||$. Reconfiguration speed is the distance between the matrix *C* ( *t* )  and the matrix at a previous time point *Δ*, so *s* ( *t* ) = | | *C* ( *t* ) − *C* ( *t* − *τ* ) | | . Metastability is the standard deviation in time of the norm, thus: *meta* = *st* d ( ∥ *C* ( *t* ) ∥ ) .

The unified theoretical framework that expresses all the types of matrices in terms of allows to compute the time varying EVD of *C* ( *t* ) , using the Temporal Covariance EVD algorithm (TCEVD, see The Temporal Covariance EVD algorithm for the computation of dFC matrices and their eigenvectors). Thus, instead of computing *C* ( *t* )  for each time point, DySCo proposes to compute its time-varying decomposition *EVD* ( *t* ) . Then, all the DySCo measures can be computed from the matrices expressed with their eigenvalues and eigenvectors.

**Fig 2:**
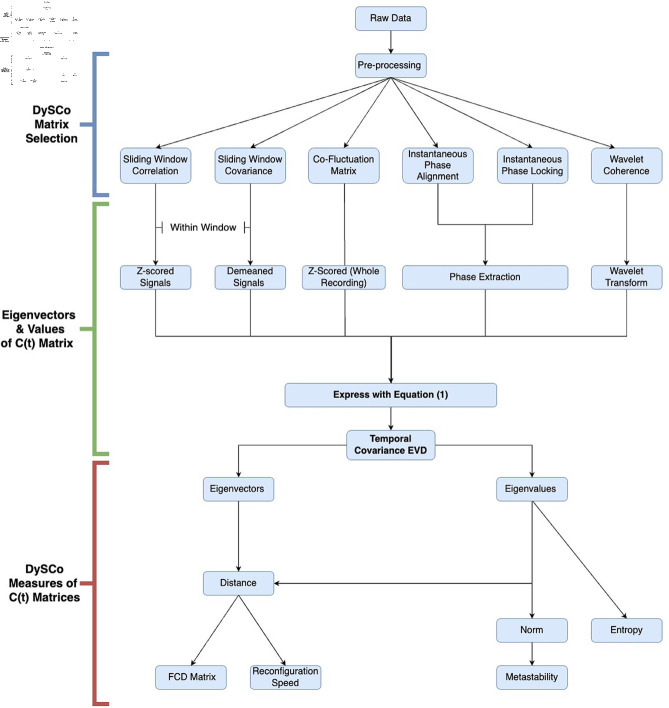
Summary of the DySCo Pipeline. This schema illustrates the main steps involved in the DySCo framework as well as important methodological decisions that must be made when using the framework. After input of raw data and appropriate pre-processing there are multiple dFC matrices as described in Theory (The DySCo theory). Based upon the choice of dFC matrix, which we define as *C* ( *t* ) , subsequent processing steps are employed (such as window size adjustment or extraction of phase) to express these dFC matrices into a single equation (). We next calculate the eigenvalues and eigenvectors associated with the dFC matrices using the Temporal Covariance EVD. The eigenvalue-eigenvector representation contains all the information needed to perform the dFC analyses, and to compute the DySCo measures described in Theory. The three main measures are Norms, Distances, and Entropy (see DySCo measures). From them we can obtain derived measures: from the norm it is possible to compute metastability (see Norm metastability), from the distance it is possible to compute the FCD matrix and the reconfiguration speed (see Distances between dFC operators).

The pipeline of the DySCo framework is implemented as packages in both MATLAB and Python and involves the following 3 steps (illustrated in Fig 2):

1 Selection of dFC matrix *C* ( *t* )  to compute from the signal of choice, it is worth noting that the framework is agnostic to the data type.

2 Running the TCEVD Algorithm described in Theory to calculate the eigenvectors and eigenvalues of the matrix evolving in time. Thus, expressing each time point as its EVD/spatio-temporal pattern.

3 Since the EVD contains all the information about the matrix, it is possible to use that to compute the DySCo measures, which can be used to characterise the evolution of the spatio-temporal dFC patterns.

### DySCo analyses are ultra-fast and memory efficient compared to previous dFC analyses

Instead of computing the *n* < 5 dimensional *C* ( *t* )  for each time point, DySCo computes the EVD of *α* = 2, without the need of *α* = 2, starting directly from the *N* dimensional data. This is done by applying the Temporal Covariance EVD algorithm (The Temporal Covariance EVD algorithm for the computation of dFC matrices and their eigenvectors). Then, the DySCo measures defined in the Theory (see The DySCo theory) can be computed directly from the EVD.

The TCEVD algorithm offers an extremely significant speed-up compared to computing the matrix and running a “naïve” EVD algorithm on it (see Investigation of computational efficiency of the TCEVD in the DySCo framework).

**Fig 3:**
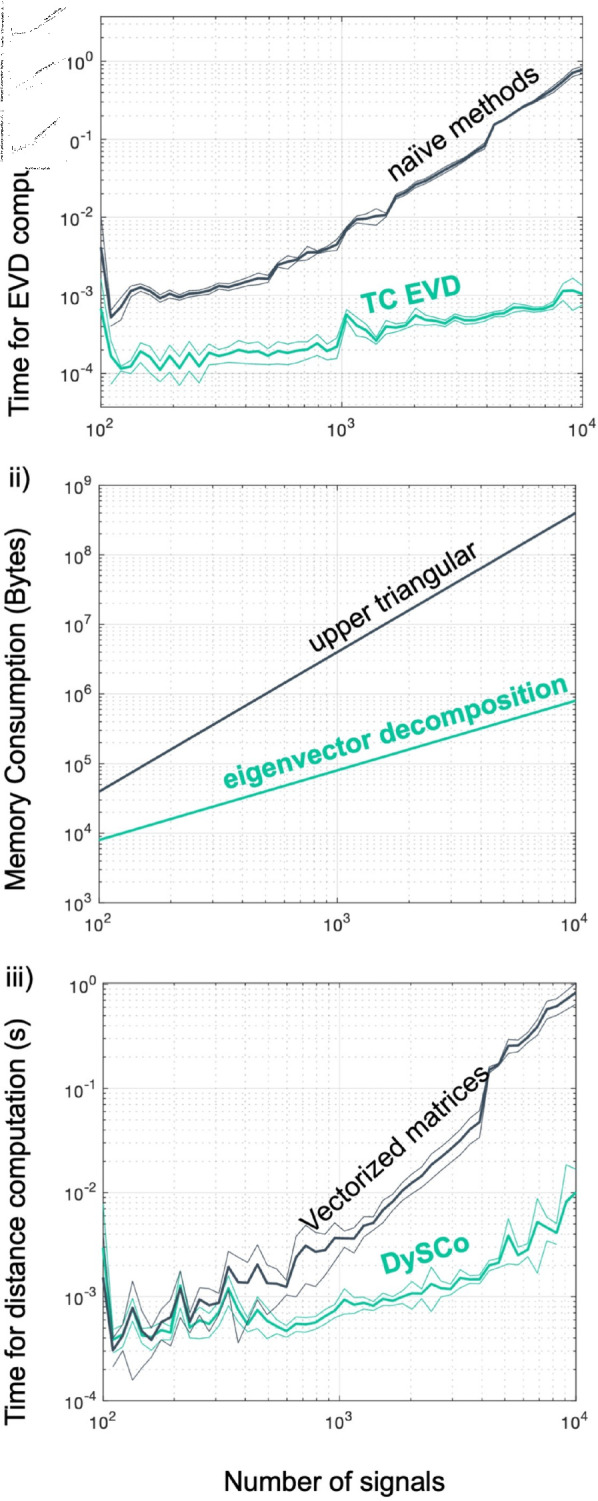
Computational efficiency of the DySCo framework. i) Comparison of computational speed of the TCEVD algorithm compared to naïve numerical methods (the MATLAB *eigs* function, see Investigation of computational efficiency of the TCEVD in the DySCo framework), using randomly generated covariance matrices in a window of size 10. We repeated the experiment 20 times. Thick lines represent the mean computation time, thin lines the  ±  variance. ii) Comparison of the memory requirements (in bytes) for the storage of the matrices using their upper triangular form (*N* ( *N* − 1 ) ∕ 2), versus using the eigenvector decomposition (*NT*). iii) Comparison of the time required to compute the Euclidean distance between two vectorized matrices versus using the DySCo EVD approach.

As shown in Fig 3 i), the TCEVD outperforms naïve numerical methods. It is 100 times faster for matrices with dimensions of 10001000, and 1000 times faster for matrices larger than 10,00010,000. As shown in Fig 3 ii), the matrix decomposition in the *T* eigenvectors also allows to save each matrix with *NT* elements instead of *N* ( *N* − 1 ) ∕ 2, offering a significant advantage in terms of RAM requirements, without loss of information.

The practical implication of this speed-up is that, since the DySCo measures on *C* ( *t* )  can be computed from its EVD without loss of information (see DySCo measures), our proposed “EVD first” approach offers a speed-up also on the DySCo measures and thus on the dFC analysis.

To offer an example, we compare our approach with the previous approaches to compute the similarity between two FC matrices. What is typically performed both in dFC and in “static” FC is to compute the Euclidean Distance (or Pearson’s R) between the vectorized matrices. This is done to perform clustering of dFC states [2, 4, 42], to compute the evolution of states in time [13, 43] or to do fingerprinting of functional connectivity matrices [44, 45]. In the Theory we show that this quantity can be obtained by the Distance 2 (see Distances between dFC operators). In Fig 3 iii) we compare this approach (compute the matrices, vectorize them, compute the distance) with the DySCo approach (compute the EVD, use the DySCo Distance 2). Fig 3 ii) and iii) confirm that the DySCo approach is up to 100 ×  faster and more memory efficient than computing the vectorized matrices.

### Application to a simulated dataset

**Fig 4:**
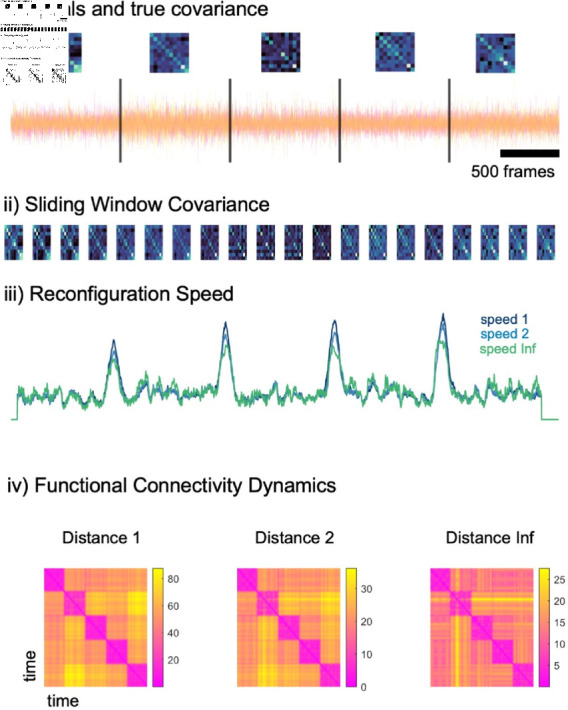
Application of the DySCo measures to a simulated dataset. i) N=10 simulated signals and the five underlying covariance patterns, corresponding to brain states. ii) The sliding window covariance matrix computed using the DySCo formula. iii) Reconfiguration speed with a lag of 100 frames shows peak corresponding to the switches between brain states (the three colors are the three options to compute distance as defined in the theory, see Distances between dFC operators). iv) Functional Connectivity Dynamics matrices. $FCD_{ij}$ is the distance between the matrix at time $t_{i}$ and the matrix at time *t_j_* using the three possible distances proposed in the DySCo framework.

We first ran DySCo on a simulated timeseries: N=10 random signals with a time-varying underlying covariance matrix (see Fig 4, see Application to simulated data). We computed the time-varying EVD of the matrices and then the Reconfiguration Speed, which is the distance between a matrix *C* ( *t* )  and a matrix at a previous time point *C* ( *t* − *τ* )  and thus quantifies the speed of the evolution of the time-varying matrices in time (see Distances between dFC operators):


$$\begin{array}{c} s(t)=dist(C(t),C(t-\tau )) \end{array}$$ (2)

The reconfiguration speed shows peaks coinciding with the switches from one matrix to the other. We also computed the Functional Connectivity Dynamics (FCD) matrix, which is the matrix of all the possible distances between *C* ( *t* )  at different time points (Distances between dFC operators), measuring the pointwise similarities between spatial connectivity patterns across time:


$$\begin{array}{c} {FCD}_{ij}=dist(C(t_{i}),C(t_{j})) \end{array}$$ (3)

The Functional Connectivity Dynamics confirms that the timeseries is temporally clustered in the 5 planted patterns. Altogether, these results show that the dFC matrices computed with the Temporal Covariance EVD formulas match the ground truth connectivity patterns and that DySCo’s dynamic measures can quantify their changes in time.

### Application of voxel-level dFC to the HCP dataset

We used the DySCo framework to perform a dFC analysis of fMRI data. To showcase its computational efficacy we applied it to voxel-level, non parcellated signals. We used a sample of pre-processed task-based (working memory N-Back) fMRI data from the Human Connectome Project (HCP)(S1200) [46].

#### Exploration of different dynamic matrices.

Since the DySCo framework contains multiple types of dynamic connectivity matrices (sliding window correlation, covariance, co-fluctuation, phase alignment based measures and more), we first show how they behave on two example signals. For a theoretical explanation of the different matrices and their role, see the Theory (The DySCo theory).

**Fig 5:**
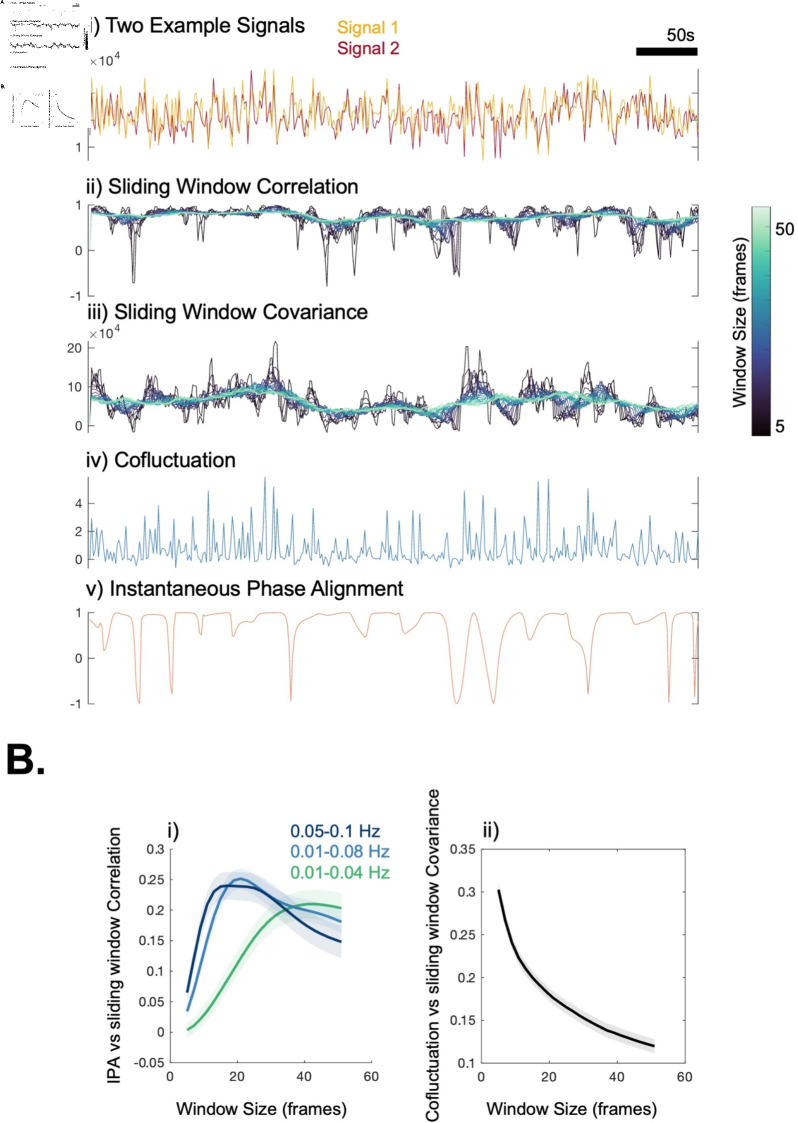
Illustration of different dynamic matrices. A: i) We selected two random signals from an example participant and plotted the timecourse of all the measures of the DySCo framework: ii) sliding window correlation, with window sizes ranging from 5 to 50; iii) sliding window covariance, with window sizes ranging from 5 to 50; iv) co-fluctuation; v) instantaneous Phase Alignment. B: i) The average (across different couples of signals, across subjects) correlation in time between instantaneous Phase Alignment and sliding window correlation as a function of the window size. ii) The average (across different couples of signals, across subjects) correlation in time between co-fluctuation and sliding window covariance as a function of the window size.

Looking at Fig 5A, the sliding window correlation matrix and the covariance matrix convey two different pieces of information, the latter being more sensitive to amplitude changes. As expected, a larger window size (in lighter shades) implies a suppression of high amplitude time-localised correlation/covariance peaks, therefore, losing time sensitivity. As described in the Theory (see Mathematical structure of the DySCo dFC matrices), the instantaneous Phase Alignment (iPA) value is similar to a correlation of signals approximated by sinusoids at a specific bandwidth of observation. Indeed, in Fig 5B i), we see a peak at a specific timescale where the iPA is most similar to the sliding window correlation. We also find that this peak depends on the bandwidth of the signal: a lower bandwidth shifts the peak to the right. Indeed, the more low-frequency the phase alignment, the larger the window needed to capture this with a correlation matrix. Moreover, as quantified in Fig 5B ii), the co-fluctuation is similar to a covariance matrix with window size approaching 1.

This procedure was also used as a tool to investigate and choose the matrix for the HCP application. We chose the sliding window correlation matrix based on the following considerations:

1) For the purpose of this work, coordinated variation was more relevant than intensity, so we excluded covariance and co-fluctuation.

2) As explained in the Theory, and confirmed by this section, matrices based on instantaneous phases assume a specific, narrow, bandwidth of observation of the signals (timescale), and, moreover, assume that brain areas are modelled as sinusoids. We did not have justifications for these two hypotheses in our signals, which is why we chose the sliding window correlation matrix.

### DySCo analysis of the HCP dataset

We computed a voxelwise sliding window correlation matrix with a window size of 21 frames. Matrices were denoised by retaining their 10 first eigenvectors.

**Fig 6:**
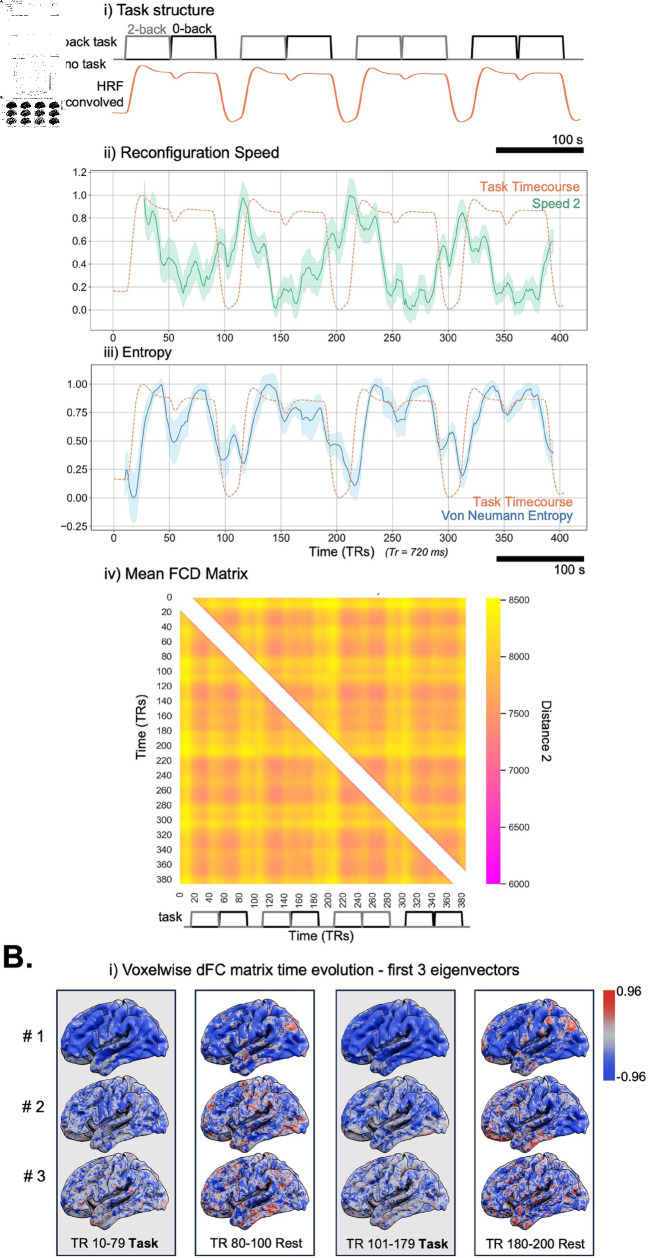
Application of DySCo to HCP dataset (all subjects). A: i) The task structure (gray line), and the HRF convolved task timecourse, in orange (see HCP task fMRI data). ii) Shows the mean reconfiguration speed (green) standard error (shaded) calculated from the obtained eigenvalues across 100 subjects with a window size of 21. The dashed line again shows the task timecourse of the HCP n-back task (r = –0.46, p  <  0.001). iii) Shows the mean von Neumann Entropy (blue) standard error (shaded) calculated from the obtained eigenvalues across 100 subjects. The dashed line shows the Task timecourse of the HCP n-back task (r = 0.76 , p  <  0.001). iv) Shows the FCD matrix averaged across all subjects. The entry *ij* of the FCD matrix (see Distances between dFC operators) represents the distance 2 between the dFC matrix at time $t_{i}$ and the dFC matrix at time $t_{j}$. B: To give an example of evolution in time of the sliding window correlation matrices, we show them by using their first 3 eigenvectors (averaged across all subjects). We display the first half of the recording to maximise space for brain rendering.

As in Fig 6, the reconfiguration speed for all subjects shows the same distinctive peaks as was observed in the simulated data with changes in task, with the largest peaks shown at the onset of task switching (rest/task) indicating shifts in connectivity patterns between task and rest.

The average Von Neumann entropy shows a positive correlation with the task timecourse (r = 0.76 , p  <  0.001). In addition, the averaged FCD matrix across all subjects shows a block structure corresponding to the task timecourse. In Fig 6B, we visualise the extremely high-dimensional sliding window correlation matrix (almost 1 billion entries, impossible to show as a matrix) by means of its 3 leading eigenvectors (plotted on the FSLR projection (32K MSM)). We show how the matrix changes at different moments of the recording.

**Fig 7:**
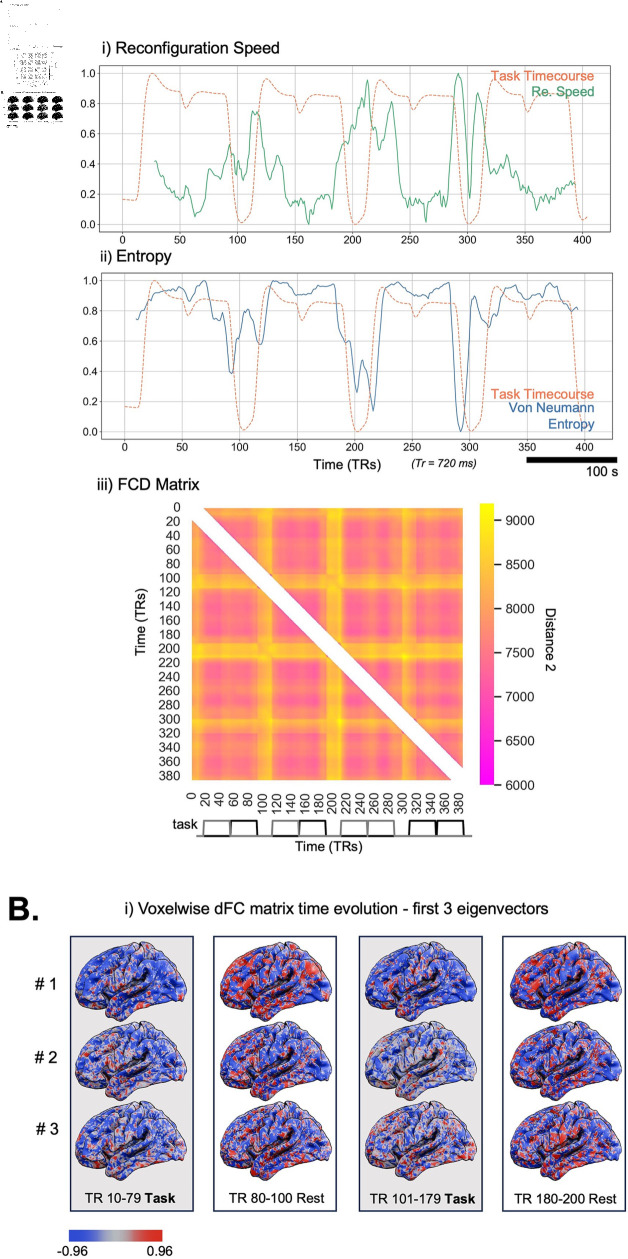
Application of DySCo to HCP dataset (One Example Subject). A: i) Shows the reconfiguration speed (green) for the single example subject. The dashed line shows the Task timecourse of the HCP n-back task (r = –0.66, p  <  0.001). ii) Shows the von Neumann Entropy (blue). The dashed line shows the Task timecourse of the HCP n-back task (r = 0.89, p  <  0.001). iii) Shows the FCD matrix for the single example subject. B: To give an example of evolution in time of the sliding window correlation matrices, for the single example subject, we show them by using their first 3 eigenvectors. We display the first half of the recording to maximise space for brain rendering.

In Fig 7 we also show the same measures applied to a single, example, subject using the same parameters and matrix choices. The reconfiguration speed here also shows peaks at the onset of switching from rest to task, but also distinctive peaks during the 2-back task. The Von Neumann entropy measure for a single subject also shows a positive correlation with the accompanying task timecourse (r = 0.89, p  <  0.001). The FCD matrix also presents a distinctive block structure corresponding to task time-course.

We chose the correlation of Von Neumann Entropy with task timecourse as a measure to test the sensitivity of our proposed approach to the change of window size. S1 Fig shows the timecourse of the Von Neumann entropy using multiple window sizes ranging from 10-24 frames. This shows that our results are robust to a change in window size. The correlation between entropy (at specific window size) and task timecourse is also shown and varies between a min of 0.69 (window size = 10) and a max of 0.87 (window size = 19).

## Discussion

We bring the most commonly used dynamic functional connectivity (dFC) methodologies [2, 4, 13, 17, 34] into an integrated mathematical and analysis framework. This enables the definition of a set of metrics, associated with the eigenvectors and eigenvalues of the dynamic matrices, that have a common underpinning and interpretation. At the core of the framework lies the Temporal Covariance EigenVector Decomposition (TCEVD) algorithm which allows an extremely fast eigendecomposition of ultra-low-rank matrices. This is made possible by using only the eigenvectors corresponding to non-trivial eigenvalues, thus retaining all the dFC information and allowing the computation of all the dFC measures without the need to explicitly reconstruct the matrices themselves [15]. This opens the way to both the analysis of extremely high-dimensional data and real-time applications, which up to now have been considered prohibitive. For example, explicitly computing the sliding window correlation matrix for a matrix containing 900M elements - as with the HCP data - would be too computationally intensive for most PCs.

### The DySCo view on brain dynamics

We believe that the extremely fast computing capacity offered by the TCEVD taken together with the DySCo measures (DySCo measures) and the considerations made in the Theory section (The DySCo interpretation of dFC) pave the way for using dFC as a different tool to examine brain dynamics.

A reasonable starting point to study the dynamics of a system in a data driven way is to identify its statistical regularities [26]. However, as argued in [47], the simplest explanation is not necessarily the best. For example, PCA provides a single covariance pattern for a whole dataset, but as pointed out in Fig 1, these axes of variation may change through time, as the system is not in a stationary state. PCA thus fails to capture dimensions and variations that have a physiological meaning. DySCo provides the capacity to investigate how these axes evolve: instead of defining a unique, static set of dimensions, we can study how the axes themselves change with time. This introduces a different point of view on dynamic signals, which does not look at their trajectories in a single space, but rather looks at how the space itself changes in time: we directly characterise the spatio-temporal patterns of interaction, similarly to the approaches in [17, 48].

We have shown that this view is complementary to the study of how region-wise connections change. The study of dFC requires few assumptions on the signals, and the measures we introduced go at the core general properties of statistical patterns of interaction: their spectral features.

Thus, we believe that DySCo is a timely translational tool that provides high-speed computing capacity that can treat the increasing dimensionality of data generated by new modalities such as from widefield calcium imaging and kilo-scale electrophysiology [40, 41]. This will enable a uniform treatment of dFC across species and fields, since the core interaction properties of the signals and their evolution in time do not depend on the specific features of the signals, and thus may be preserved across taxonomies (see [25, 49]).

We have also shown that the DySCo view is complementary but not antithetic to the “classic” dFC view [2, 27]. Indeed, the measures proposed in the DySCo framework allow for all the typical analyses in dFC, i.e., quantification of time-varying total connectivity (e.g. [50]), quantification of the temporal structure of the evolution of dFC matrices [13], metastability [37], clustering [4, 17] (see DySCo measures). This can be done in a unified and computationally optimized manner. We note also that the DySCo framework and measures can be extended also to other dFC matrices in the future, as long as they are symmetric (we have provided a general result, see Mathematical structure of the DySCo dFC matrices) - such that the spectral theorem holds and the DySCo measures can be computed.

### Discussion of the DySCo matrices, the DySCo measures and their related parameters

We now briefly discuss the similarities and differences of the various dFC methods considered, and the main parameter choices to make in the DySCo analysis. An advantage of the DySCo framework is that it depends on very few design choices. The most important are the matrix type and the window size (if required). The other choices to be made are related to the norms and distances used, which might be used either for sensitivity analyses, or dependent on experimental settings, task or imaging modality. Another aspect of the pipeline that can be tuned is the number of eigenvectors used to approximate the dFC matrix. The framework as presented here makes a lossless representation of the information possible, but one can choose to focus on a subset of non-trivial eigenvectors. This can be used as a denoising method, if one considers that the information contained in the leading eigenvectors captures the essential signal information [17].

In our application, we chose our parameters based upon exploration of the parameter space, and consistency with the plethora of existing literature on parameter optimisation in these data types [51]. However, it should be clear that the parameters chosen in this analysis pipeline are specific to the data used and are not directly transferrable to other fMRI datasets or imaging modalities, such as EEG/MEG/wide-field calcium, and may be dependent on the experimental design.

As expected, our results confirm that changing the window size for the sliding window correlation and covariance approaches changes the temporal scale of observation [2]. Indeed, with small window sizes it is possible to see high amplitude peaks, which get suppressed at larger window sizes. Our results further suggest that the covariance matrix captures the co-fluctuation matrix in the limit of the window size approaching 1, which makes it very sensitive to the co-fluctuation peaks [30]. The physiological relevance of these peaks and their interpretation depends on the experimental setting, e.g. TR, and is left to experimenter.

As for the relationship between the instantaneous Phase Alignment (**iPA**) approach and the sliding window correlation, we found that the instantaneous Phase Alignment is sensitive to a specific (narrow) time-scale, which is specified by the bandwidth of observation, confirming the observations made in [15, 17, 34, 37]. Indeed, shifting the frequency of observation shifts the window size at which the maximal similarity with the sliding window correlation was observed. Therefore, when using the **iPA** matrix, the experimenter should be mindful of the bandwidth they are selecting and the implications this has on the signal that is used.

### Evidence from the HCP data

We have shown that the metrics introduced are sensitive to the changes in the functional connectivity patterns associated with the task, confirming that dFC fluctuations are genuine and not spurious, which is a debated topic in the field [29].

The reconfiguration speed quantifies the speed of the evolution of the brain states (expressed by **C(t)**) in time [13]. A spike in reconfiguration speed (Figs 6 and 7) reveals a rapid modulation in functional connectivity patterns associated with switching between task and rest. The observed smaller spikes describe changes in functional connectivity patterns between different task types. These results hold true also at the individual subject level. While it is not suitable to draw inferences as to the meaning of each of these peaks in relation to tangible connectivity patterns, it is clear that this measure is capable of effectively capturing both changes during an activity and between activities. The FCD matrix is also able to summarise across the similarities and differences in connectivity patterns associated with tasks across the whole recording. We observe a distinctive tiled structure that reflects the block task design. During task, all the dFC matrices are more similar distancewise, while rest is associated with periods where the dFC reconfigures itself.

As described in the Theory section, the Von Neumann entropy can be seen as a measure of how broadly the brain explores different axes of variation and is thus related to the dimensionality of the data, as discussed in Von Neumann entropy of the dFC pattern and its interpretation. (see also [38]). The obtained measures of entropy demonstrate that the eigenvalue spectrum is informative about the dFC matrix structure and that it changes during task. The observed increase in entropy during task represents a dispersion of the eigenvalue spectrum, which indicates increased dimensionality. This may be explained by the fact that during rest the brain is characterised by a dominance of the default mode network (DMN) [52], which causes the eigenvalue spectrum to be more peaked.

## The DySCo theory

This section starts from known linear algebra facts to build a theory for dynamic FC matrices. From now on, we will refer to any matrix representing dynamic Functional Connectivity (dFC matrix) as *C* ( *t* ) .

DySCo proposes a single unified framework to compute *C* ( *t* )  matrices, their eigenvectors, and metrics of interest: the norm of the matrix *C* ( *t* )  (see Norms), which is related to the amount of instantaneous interactions, the distance between *C* ( *t* )  matrices (Distances between dFC operators), to perform clustering or to analyse how spatio-temporal patterns change in time, and the *C* ( *t* )  entropy, which is related to how multidimensionally the signals explore their space (Von Neumann entropy of the dFC pattern and its interpretation.). The DySCo framework is based on the Temporal Covariance EVD (TCEVD), which is orders of magnitude faster for dFC applications compared to brute force algorithms. DySCo unifies the treatment of different dFC matrices: co-fluctuation, phase locking, phase alignment, correlation, covariance matrices and more [15]. We developed the code to compute all DySCo quantities, available as packages in both MATLAB and Python at *https://github.com/Mimbero/DySCo*.

### Mathematical structure of the DySCo dFC matrices

We will show that all dFC matrices have the same structure and can therefore be treated within the same mathematical framework. Given a multivariate time series of dimension *N*, all of the above matrices can be expressed as a dyadic sum, i.e.:


$$\begin{array}{c} C(t)=\overset{t+T∕2}{\underset{i=t-T∕2}{\sum }}w_{i}x(i)x{\left(i\right)}^{⊤} \end{array}$$ (4)

where the vectors *x* ( *i* )  are a representation of the signals at a time *i*, see S1 Appendix for the full derivation. Given this structure, the *C* ( *t* )  matrices are:

- symmetric
- positive-semidefinite
- low-rank: the rank is not larger than *T*
- of fixed trace, which is equal to the number of signals *N*. This property holds only for a subset of *C* ( *t* )  matrices: the sliding window correlation matrix, the “tapered” window correlation matrix, the “instantaneous Phase Alignment” matrix, the Phase Locking matrix; see below.

We now explicitly write the dFC matrices *C* ( *t* )  in the format of . Please see Table 1 for a summary of their properties.

**Table 1:** Description of the dFC matrices presented above and their main properties.

	Sliding Window Covariance	Sliding Window Correlation	Co-Fluctuation	instantaneous Phase Alignment	Phase Locking
Meaning	Covariance in a window of T frames	Correlation in a window of T frames	Pairwise product of signal amplitudes at time t	If signals are modeled as oscillations, measures to what extent they are instantaneously in phase or antiphase	Complex value indicating if signals keep a phase difference in a window of T frames
Meaning of eigenvectors	Main axes of variation of the signals. It is equal to a sliding window PCA.	Main axes of variation of the normalised signals (correlation is a normalised covariance)	They are the signals themselves at time t	Modes of co-oscillation at the frequency of observation	It is like performing a PCA on the complex exponentials of the signal phases instead of the signals
Rank (= n of non-null eigenvalues)	T-1	T-1	1	2	T
Trace (= sum of eigenvalues)	variable	= N of signals	= first and unique non-null eigenvalue	= N of signals	= N of signals
Notes	Windows can be smoothed/tapered	Windows can be smoothed/tapered. Works also with Spearman correlation or partial correlation	The eigenvalue is equal to the Euclidean norm of the array collecting all signals at time t. Having rank 2 and fixed trace, the first eigenvalue contains all information about the spectrum	Having rank 2 and fixed trace, the first eigenvalue contains all information about the spectrum	If the window size is 1, its real part is the instantaneous Phase Alignment matrix.
			In this case the TCEVD algorithm is trivial since the eigenvectors are arrays themselves	There is an analytical formula for the EVD	If phases are computed using the Wavelet transform, with some simple manipulations this becomes the Wavelet Coherence.

- **Sliding window Correlation Matrix (Pearson/Spearman/Partial)**: In this approach, correlations are computed in a window of size *T*:
  $$\begin{array}{c} C(t)=\frac{1}{T-1}\overset{i=t+T∕2}{\underset{i=t-T∕2}{\sum }}w_{i}z(i)z{\left(i\right)}^{⊤} \end{array}$$ (5)
  Where *z* ( *i* )  are the z-scored signals in the window  [ *t* − *T* ∕ 2 , *t* + *T* ∕ 2 ] . This is the most commonly employed matrix *C* ( *t* )  [25, 53], together with the sliding window covariance matrix [4, 54]. Windows can be “square” if $w_{i}=1\;\forall \;i$ or “tapered”/“weighted” if the weights *w* form a window with smooth edges [2]. applies also to Partial Correlations and Spearman Correlations, as long as the signals are previously transformed (see S1 Appendix S1.1.1 for details).
- **Sliding window Covariance Matrix**: Similarly to the correlation case, the covariances are computed in a window of size *T* with weights $w_{i}$, *y* ( *i* )  are the demeaned signals in the window.
  $$\begin{array}{c} C(t)=\frac{1}{T-1}\overset{i=t+T∕2}{\underset{i=t-T∕2}{\sum }}w_{i}y(i)y{\left(i\right)}^{⊤} \end{array}$$ (6)
- **Co-fluctuation Matrix**: The co-fluctuation matrix can be conceived as a sliding window covariance matrix with window-size *T* = 1:
  $$\begin{array}{c} C(t)=\zeta (t)\zeta {\left(t\right)}^{⊤} \end{array}$$ (7)
  where *ζ* is the z-scored signal in the whole recording. It holds the property that the static correlation matrix is the average of all the instantaneous co-fluctuation matrices [30].
- **instantaneous Phase Alignment Matrix**: The instantaneous Phase Alignment Matrix (**iPA**)[15, 17, 55] quantifies the extent to which signals are instantaneously in phase/anti-phase. The Phase Alignment Matrix assumes that every brain area can be modelled as an oscillator with an instantaneous phase *θ* ( *t* ) . This is valid for narrowband signals, like EEG or fMRI filtered in a specific band. The extraction of the instantaneous phase requires the use of the Hilbert transform [15, 34] or wavelet transform [56]. The “instantaneous Phase Alignment” measures the cosine of the phase difference of the signals. The vectors *c* ( *t* )  and *s* ( *t* )  are respectively the element-wise cosine and sine of the instantaneous angles [15, 18]. It is worth noting that the matrix *iPA* ( *t* )  is analogous to the correlation matrix under the local approximation of signals with sinusoids, see S1 Appendix. Therefore, the matrix *iPA* ( *t* )  has the same role as the sliding window correlation matrix. However, the sliding window computes correlations in a window, while the matrix *iPA* ( *t* )  assumes that the signals are locally approximated by simple oscillations.
  $$\begin{array}{c} C(t)=c(t)c{\left(t\right)}^{⊤}+s(t)s{\left(t\right)}^{⊤} \end{array}$$ (8)
- **Sliding Window Phase Locking Matrix**: the Phase Locking Matrix (**PL**) quantifies if there is a constant phase delay of the signals in the window [57]. It is the complex counterpart of the **iPA** matrix. It also requires to extract the instantaneous phases of signals:
  $$\begin{array}{c} C(t)=\frac{1}{T}\overset{\tau =t+T∕2}{\underset{\tau =t-T∕2}{\sum }}\text{exp}(i\theta (\tau ))\text{exp}{\left(i\theta (\tau )\right)}^{H} \end{array}$$ (9)
  where $\text{exp}(i\theta (\tau ))=\left[\begin{array}{c} e^{i\theta_{1}(\tau )}\\ e^{i\theta_{2}(\tau )}\\ \vdots \\ e^{i\theta_{N}(\tau )} \end{array}\right]$ is the vector of the element-wise complex exponentials of the phases of the signals and *H* denotes its hermitian conjugate.
- **Wavelet Coherence Spectrum**: the Complex Wavelet Coherence Matrix (**CWC**) quantifies the coherence between signals based on their wavelet transforms [58, 59]. It requires computing the wavelet transform of the signals and their complex coefficients:
  $$\begin{array}{c} C(t)=W_{f}(t)W_{f}{\left(t\right)}^{H} \end{array}$$ (10)
  where $W_{f}(t)=\left[\begin{array}{c} \frac{w_{f,1}(t)}{\left||w_{f,1}(t)|\right|}\\ \frac{w_{f,2}(t)}{\left||w_{f,2}(t)|\right|}\\ \vdots \\ \frac{w_{f,N}(t)}{\left||w_{f,N}(t)|\right|} \end{array}\right]$ is the complex vector of normalised wavelet coefficients of the signals at time *t* and frequency *f*. Similarly to the Phase Locking matrix, these values can be also averaged in a window [58, 59].
- **General form of DySCo valid matrices**: we show in the Appendix (see S1 Appendix) that can be extended to any matrix of the type
  $$\begin{array}{c} C_{ij}(t)=\underset{\tau \in \Omega (t)}{\sum }f(x_{i}(\tau ),x_{j}(\tau )) \end{array}$$ (11)
  where the function *f* is of the form:
  $$\begin{array}{c} f(x_{i}(\tau ),x_{j}(\tau ))=\varphi (x_{i}(\tau ))+\varphi (x_{j}(\tau ))+\psi (x_{i}(\tau )-x_{j}(\tau ))+\underset{k}{\sum }\pi_{k}(x_{i}(\tau ))\pi_{k}(x_{j}(\tau )) \end{array}$$ (12)
  where *φ* and $\pi_{k}$ are generic functions, while *ψ* is an even function that is periodic or has a finite support.
  This includes all the matrices above, and more, like the matrix of squared Euclidean Distances, where $\varphi (x)=x^{2}$, *ψ* = 0, and $\pi (x)=\sqrt{2}i\;x$. This also includes any periodic function of the phase difference *Δθ* between two signals.

### The DySCo interpretation of dFC

Dynamic Functional Connectivity is usually conceived as study of the changes in the connection strength between nodes in a brain functional network (irrespective of the presence or absence of an anatomical circuitry) [2, 60]. Practically speaking, this involves computing the dynamic matrix *C* ( *t* )  and performing operations and measures on *C* ( *t* ) , for example, clustering.

This is instead the DySCo interpretation of dFC: shows that *C* ( *t* )  is a statistical representation of spatial patterns at a time *t* estimated over a time window of size *T* (in case of **iPA** and co-fluctuation, *T* = 1). The evolution of *C* ( *t* )  therefore represents the evolution of the spatio-temporal patterns: i.e., a low-dimensional geometry “navigating” a high-dimensional space. The size of the window determines the trade-off between the accuracy of the estimation of the statistical pattern and the resolution in time. In general, the choice of the window depends on the properties of the signals and cannot be determined a-priori.

This interpretation has the following implications:

- *C* ( *t* )  is a way to study the dynamics of a complex, multidimensional system in a data-driven way. Most complex systems move in a landscape of different spatio-temporal interaction patterns [61]. As the system explores different states (low-dimensional object in a high-dimensional space), *C* ( *t* )  expresses how this low-dimensional embedding changes with time (see Fig 1 for a toy example).
- As all *C* ( *t* )  are symmetric matrices, the most natural way to quantify this low-dimensional embedding is their eigendecomposition. And, since they are low-rank matrices, a small number, Rk ( *C* ( *t* ) ) ≤ *T* ≪ *N*, of eigenvectors suffices to express all the information contained in the matrix:
  $$\begin{array}{c} C(t)=\overset{\text{Rk}(C(t))}{\underset{i=1}{\sum }}\lambda_{i}(t)u_{i}(t)u_{i}{\left(t\right)}^{⊤} \end{array}$$ (13)
- Using the “connectivity matrix” view, one could argue that for a too small window, *C* ( *t* )  is able to capture neither true correlations/covariances/phase locking patterns, nor the underlying network structure. Instead, the “eigenvector” view posits that the eigenvectors, regardless of the size of the window, are the principal axes of variation in the data. Even in a 1 time frame window, the main axis of variation of the signal is the signal itself. In addition, the eigenvector representation of *C* ( *t* )  allows a straightforward strategy to denoise the signal, by retaining a subset of eigenvectors associated to the largest eigenvalues, which are proportional to the amount of variance expressed.
- A third possible view, that we cite here for completeness, is that *C* ( *t* )  can be seen as an average of pairwise products, as suggested by [25], thus we can interpret *C* ( *t* )  as the second-order Taylor expansion of any function of brain data. Indeed, we can formally write any brain output, for example behaviour, as *b* ( *t* ) = *f* ( *x* ( *t* ) ) , where *x* ( *t* )  is brain activity and *f * a function mapping activity to behaviour. Using a second-order Taylor expansion in $x(t_{0})$, we can write $f(x(t))\approx f(x(t_{0}))+\sum_{i}\frac{\partial f}{\partial x_{i}}{|}_{t=t_{0}}(x_{i}(t)-x_{i}(t_{0}))+\frac{1}{2}\sum_{i}\sum_{j}\frac{\partial^{2}f}{\partial^{2}x_{i}x_{j}}{|}_{t=t_{0}}(x_{i}(t)-x_{i}(t_{0}))(x_{j}(t)-x_{j}(t_{0}))+∊$. Overall, this suggests that *C* ( *t* )  can be seen as the quadratic term of a second-order approximation of brain function (see [25] for the full development).

### On the importance of the Leading Eigenvector

The Leading Eigenvector is associated to the largest eigenvalue of *C* ( *t* )  and is widely employed in both phase-locking studies [12, 17, 18] and correlation studies (in this case also known as Eigenvector Centrality -[62]). Its importance can be summarised as representing the best single-vector approximation of a dFC matrix:

- It is the main axis of variation of the signals in the window/bandwidth of observation (dominant mode of dynamic connectivity). Each of its entries refers to an anatomical location, so the leading eigenvector is useful for visualising the dominant mode in the anatomical space [63].
- The outer product of the leading eigenvector with itself approximates *C* ( *t* ) . Indeed, if $C(t)=\sum_{i=1}^{\text{Rk}(C(t))}\lambda_{i}(t)u_{i}(t)u_{i}{\left(t\right)}^{⊤}$, then $C(t)\approx \lambda_{1}(t)u_{1}(t)u_{1}{\left(t\right)}^{⊤}$. This is also used to measure “eigenvector centrality”, because it is a measure of how “central” an area is (i.e. how much it engages interactions with the others). In the anatomical space, an intuitive way to read the leading eigenvector is the following: areas with the same sign have a positive interaction (i.e. positive correlation, covariance, etc), while areas with opposite signs have a negative interaction. The amplitude of the leading eigenvector indicates the centrality of the area.
- Following the same logic, the leading eigenvector of the **iPA** matrix can be seen as the main mode of oscillation, since the **iPA** matrix expresses co-oscillations rather than correlations. Multiple studies have shown that the brain explores a set of oscillatory modes, which can be computed by performing temporal clustering (e.g. k-means) of the leading eigenvectors of the **iPA** matrix. This is known as the LEiDA method (Leading Eigenvector Dynamics Analysis) [17, 37].

### The Temporal Covariance EVD algorithm for the computation of dFC matrices and their eigenvectors

In this subsection, we present the methodological core of the DySCo framework, which allows for ultra-fast eigendecomposition of very high-dimensional matrices.

Let us consider a generic matrix *C* ( *t* )  that is expressed as a dyadic sum $C(t)=\sum_{i=1}^{T}x(i)x{\left(i\right)}^{⊤}$. Being a matrix, *C* ( *t* )  is a linear operator from ${\mathbb{R}}^{N}$ to ${\mathbb{R}}^{N}$, where *N* is the number of signals. The fact that a dFC matrix is a sum means that all its outputs lie in the space spanned by the *T* vectors *x* ( *i* ) . This implies that its rank Rk is not higher than *T*, so Rk ≤ *T*. Indeed, the rank of a linear operator is the dimensionality of the space where it maps its inputs. Moreover, this implies that the eigenvectors of a dFC matrix must be a linear combination of the *T* vectors, and the eigenvectors associated with non-null eigenvalues will be no more than the rank, so no more than *T*. This means that any dFC matrix is an extremely low-rank operator, provided *T* ≪ *N* (which is the case in practical applications). Thus, Rk ≤ *T* ≪ *N*, and the full information of the dFC pattern can be stored without approximation, or losslessly, in at most *T* eigenvectors.

Moreover, the Rk eigenvectors and their associated eigenvalues can be computed using the Temporal Covariance EigenVector Decomposition algorithm (TCEVD) (see S1 Appendix):

- for a generic dFC matrix at a generic time point *t*, which is expressed as $C(t)=\sum_{i=1}^{T}x(i)x{\left(i\right)}^{⊤}$, we define the Temporal Covariance matrix $\text{R}{\left(t\right)}_{ij}=x{\left(i\right)}^{⊤}x(j)$. This matrix of scalar products has the size *T* × *T*
- The eigenvalues of the matrix *C* ( *t* )  are the eigenvalues of the Temporal Covariance matrix *R* ( *t* ) .
- Each eigenvector **v** of the Temporal Covariance matrix is a *T*-tuple of coefficients. The eigenvectors of the matrix *C* ( *t* )  are a linear combination of the **x** vectors, where the coefficients are $v_{i}$.

Note that the matrix *iPA* ( *t* ) , (see ), is rank 2 and therefore its associated Temporal Covariance matrix is a 2 × 2 matrix, and its eigenvalues and eigenvectors can be computed analytically. For the analytical calculations and an extended discussion on the **iPA** matrix structure, see [15]. Note also that the sliding window correlation (see ) and covariance () matrices are rank *T*–1 and not *T*, given that before application of the signals are demeaned and therefore lose a degree of freedom. Finally, note that the co-fluctuation matrix () is rank 1, since it is just $\zeta (t)\zeta {\left(t\right)}^{⊤}$, so its eigenvector is trivially *ζ* ( *t* ) , and its associated non-null eigenvalue $∥\zeta (t){∥}_{2}^{2}$.

This algorithm implies that the full information of a dFC matrix can be extracted without the need to compute the matrix itself. Its eigendecomposition (EVD) operates on the *T* × *T* Temporal Covariance matrix, with associated time complexity of $O(NT^{2}+T^{3})$ and space complexity of $O(T^{2})$ instead of a *N* × *N* one, that has associated time complexity of $O(N^{3})$ and space complexity of $O(N^{2})$ [64, 65]. This can make algorithms for computation and storage orders of magnitude more efficient (see Fig 3, in DySCo analyses are ultra-fast and memory efficient compared to previous dFC analyses). We refer the reader to the S1 Appendix for an extended proof that considers the general weighted sum $C(t)=\sum_{i=1}^{T}w_{i}x(i)x{\left(i\right)}^{⊤}$.

Finally, note that the TCEVD is efficient when *T* < *N*, which is typically the case in dFC. In case *T* > *N*, the DySCo framework still applies, however, we suggest using a classic EVD because the TCEVD would not improve the computational speed given that matrices would be full rank.

We also observe that this approach is similar to the Dynamic Mode Decomposition introduced in Fluid Mechanics [48].

#### On the meaning of the Temporal Covariance matrix.

The Temporal Covariance matrix also has a physical meaning: it is the matrix of scalar products, and thus of matching/similarity, of the spatial signals at different time points. The Temporal Covariance matrix is therefore in the time domain, and quantifies whether a couple of time points are characterized by a similar whole-brain configuration. Its eigenvectors **v**, the temporal modes, are time vectors (*i* = 1…*T*) and are associated to the changes in time instead of the changes in space of the signals. For example, the dominant temporal mode would be a dominant pattern of temporal similarity in the signals.

### DySCo measures

Here, we introduce a set of measures that are aimed at characterising the spatio-temporal patterns captured by the dFC approach and brain activity in general.

#### Norms.

The norm of a matrix *C* ( *t* )  is a synthetic measure of the overall amount of interactions expressed within [50]. Here we propose three of the most employed matrix norms in linear algebra. The TCEVD of the dFC matrices suggested in the DySCo framework allows to compute these norms without computing the matrices directly and storing them.

- The Schatten norm-1 (Trace norm) of a matrix, $∥C(t){∥}_{1}$, is the sum of the absolute values of its eigenvalues: $\sum_{i}|\lambda_{i}(t)|$
- The Schatten norm-2 (Frobenius norm) of a matrix, $∥C(t){∥}_{2}$, is the square root of the sum of the squared eigenvalues: $\sqrt{\sum_{i}\lambda_{i}{\left(t\right)}^{2}}$ which coincides with the square root of the sum of all the squared values of the matrix $\sqrt{\sum_{ij}C{\left(t\right)}_{ij}^{2}}$ since dFC is symmetric.
- The Schatten norm-*∞* (Spectral norm) of a matrix, $∥C(t){∥}_{\infty }$, coincides with the largest absolute value of its eigenvalues:  max ⁡ i {|λi(t)|}.

Note that, in the specific cases of the correlation matrix and **iPA**, the matrices *C* ( *t* )  have a fixed trace (see Mathematical structure of the DySCo dFC matrices). Consequently, their norm-1 becomes trivial and simply corresponds to their size *N*. Moreover, the co-fluctuation matrix has trivially one non-null eigenvalue (see The Temporal Covariance EVD algorithm for the computation of dFC matrices and their eigenvectors). Therefore, all the norms coincide, and in that specific case they coincide with the norm of the vector *ζ* ( *t* ) .

#### Norm metastability.

Norm Metastability is the standard deviation in time of the norm of the dFC matrix [15, 55].


$$\begin{array}{c} meta=std(∥C(t)∥) \end{array}$$ (14)

Norm metastability is a measure of variability in the exploration of connectivity patterns. It is a measure of how much the dFC matrix oscillates between time points with high norm and time points with low norm, and thus reflects simultaneous tendencies for coupling and decoupling. In [15], the standard deviation of the infinite norm has been introduced as spectral metastability [11].

#### Distances between dFC operators.

If a matrix norm exists, the distance can be computed as just the norm of the difference of two matrices. Therefore:

- distance 1 is $∥C(t_{1})-C(t_{2}){∥}_{1}$
- distance 2 is $∥C(t_{1})-C(t_{2}){∥}_{2}$
- distance *∞* is $∥C(t_{1})-C(t_{2}){∥}_{\infty }$

It may also be the case, in some applications, that before computing the distances, matrices are normalised to have all unit norm.

Defining a distance between *C* ( *t* )  matrices is useful for several reasons:

- Clustering dFC patterns is a very common approach in neuroimaging [4, 17]. To cluster it is necessary to define a distance between *C* ( *t* )  matrices.
- A distance between dFC patterns allows to compute properties of how they explore the dFC space, like the speed at which the *C* ( *t* )  pattern evolves with time. The reconfiguration speed for a delay *τ* is defined [13]:
  $$\begin{array}{c} s(t)=dist(C(t),C(t-\tau )) \end{array}$$ (15)
- A distance allows to build the Functional Connectivity Dynamics matrix (FCD). FCD is a time-to-time distance matrix: the entry *ij* of FCD is the distance of $C(t_{i})$ with $C(t_{j})$. This is a condensed portrait of the evolution and properties of dFC in the whole recording [66].
  $$\begin{array}{c} {FCD}_{ij}=dist(C(t_{i}),C(t_{j})) \end{array}$$ (16)

It is still possible to apply the TCEVD (The Temporal Covariance EVD algorithm for the computation of dFC matrices and their eigenvectors) to compute the above distances, since they are based on the eigenvalues of the matrix $C(t_{1})-C(t_{2})$. Thus, it is possible to exploit the information expressed in the eigenvectors and compute the distance without reconstructing the matrices. Note that, especially in the case of norm-2, this turns out to be orders of magnitude faster than the one computed by calculating the full matrix instead of the eigenvector representation; see S1 Appendix.

Note also that, since the correlation and instantaneous Phase Alignment matrices have all ones on the diagonal, the distance 1 in that case becomes trivially zero - it is therefore suggested in those cases to use the other two distances.

Note: it is common practice to represent *C* ( *t* )  matrices as vectorised matrices (or their vectorised upper-triangular part) given the symmetry. One could then think that the matrix distance is equal to the vector distance of the vectorised matrices. However, this is incorrect, because the norm of the vectorised matrix does not coincide with the norm of the matrix, and so does the distance. The only exception is the norm-2, where the two things coincide. However, even in that case, our proposed formulation of norm-2 is still advantageous because it speeds up computations by orders of magnitude to up to two/three orders of magnitude in practical scenarios.

Another commonly employed measure is the cosine similarity between two vectorized dFC matrices [13, 66], which quantifies the matching between two matrices. This is not a distance, but a measure of similarity.

To define a measure of matching, it is necessary to define an inner product between two matrices. We introduce the Frobenius product between two matrices $C(t_{1})$ and $C(t_{2})$:


$$\begin{array}{c} \left\langle C(t_{1}),C(t_{2})\rangle =Tr(C{\left(t_{1}\right)}^{⊤}C(t_{2})\right) \end{array}$$ (17)

The square root of the Frobenius product of a matrix with itself is its Frobenius norm. So, exactly as in vectors, it is possible to define a measure of alignment:


$$\begin{array}{c} \cos(\theta )=\frac{\left\langle C(t_{1}),C(t_{2})\right\rangle }{\sqrt{\left\langle C(t_{1}),C(t_{1})\right\rangle }\sqrt{\left\langle C(t_{2}),C(t_{2})\right\rangle }} \end{array}$$ (18)

The DySCo framework also provides a fast formula to compute this quantity (see S1 Appendix).

Finally, it may be the case that the quantity of interest is a subset of eigenvectors of the matrices $C(t_{1})$ and $C(t_{2})$, without interest in the eigenvalues. In that case a measure of alignment of the eigenvectors is needed. This measure corresponds to the Frobenius distance between the projector matrices of $C(t_{1})$ and $C(t_{2})$ (i.e. the matrices that project, respectively, on the eigenvectors of $C(t_{1})$ and $C(t_{2})$). See S1 Appendix for the full derivation.

We observe that the FCD matrix, being a distance matrix, also allows nonlinear dimensionality reduction techniques such as diffusion embedding, as done in [25].

#### Von Neumann entropy of the dFC pattern and its interpretation.

The spatio-temporal complexity of brain signals is of great biological interest as it is believed to be related to health and disease, as well as consciousness [67], sleep-wake transitions [68, 69], psychedelics [70]. We thus include the Von Neumann entropy of *C* ( *t* )  [38, 71]:


$$\begin{array}{c} E(t)=-\sum \lambda_{i}(t)log(\lambda_{i}(t)) \end{array}$$ (19)

Before applying the formula the eigenvalues are normalised such that their sum is 1.

This measures how broadly the brain is exploring the pattern of its possible configurations (i.e. how multidimensionally signals are spanning their axes of variation - eigenvectors) being a measure of variability of the eigenspectrum.

**Fig 8:**
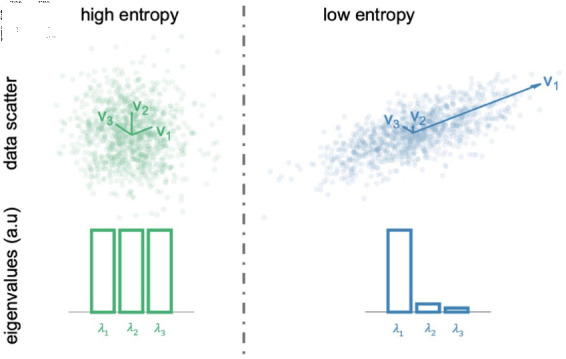
Illustration of Von Neumann Entropy. Two example cases to show the Von Neumann Entropy, in an Example 3-dimensional random signal. In the first case, there is no main axis of variation, thus the eigenvalues are all similar. This corresponds to a high Von-Neumann entropy. In the second case, there is a main axis of variation, which corresponds to a less dispersed eigenvalue spectrum. This corresponds to a low Von-Neumann entropy.

Indeed, the spectrum of eigenvalues associated with the eigenvectors expresses their relative importance. For example (see Fig 8), in case where an eigenvector is strongly dominant, the first eigenvalue is high and the others are close to zero. On the other extreme, if all the eigenvectors have the same role, meaning that the brain is exploring its configuration more broadly in the multidimensional space of all its possible configurations, the associated eigenvalues will all have similar magnitudes.

In the first limit case, the signal *x* ( *t* )  in the window would always stay parallel to itself (as a vector), so all the signals are perfectly correlated, all the signals are perfectly oscillating along the axis of variation which is *x* itself. There are no additional axes of variation. In contrast, the more the spatio-temporal patterns are rich, the more there will be axes of variation, corresponding to more eigenvectors of *C* ( *t* )  with non-null eigenvalues. For a visual explanation, see [15], where this concept is applied to the **iPA** matrix.

Note that, in case the window is weighted, the eigenvalue spectrum will be forced to make some vectors dominant. The entropy must be considered carefully in this case.

## Materials and methods

### The DySCo repository

The DySCo framework comes with a repository. We developed the code to compute all the DySCo quantities both in MATLAB and in Python, which is available at *https://github.com/* *Mimbero/DySCo*. Note that, both in MATLAB and in Python, we provide all the “core functions” (compute TCEVD, compute norm, distance, etc) to autonomously build a processing pipeline. However, we also offer an already built example Python pipeline, the one that has been used to process the HCP data. The repository also features a Python GUI to run the analyses.

### Investigation of computational efficiency of the TCEVD in the DySCo framework

We first illustrated the computational speed-up of the TCEVD algorithm expected from Theory. To do so, we fixed a window of length T=10 and generated *N* Gaussian i.i.d. random signals in the window. We computed the covariance matrix of the signals in the window using both the Temporal Covariance EVD proposed in DySCo and the standard numerical algorithm provided by MATLAB (using the *eigs* function as in the version 2021A). We computed the time needed to perform EVD in both cases. We varied *N* logarithmically, from N=100 to $N=10^{4}$. We then repeated this procedure 20 times for each *N*.

We also compared the time to compute the Euclidean distance between vectorized matrices, as in [4, 13, 42, 43, 45, 54], with the DySCo approach (use the TCEVD, then compute the distance directly from the eigenvectors). To do so, we again fixed the length T=10 and generated *N* Gaussian i.i.d. random signals in 2 windows. We computed the 2 covariance matrices for the 2 windows and then computed the Euclidean distance between the vectorized matrices. Then, we computed the TCEVD for the 2 covariance matrices and then computed the DySCo distance between them. Again, we varied *N* logarithmically, from N=100 to $N=10^{4}$. We then repeated this procedure 20 times for each *N*.

Computation times were calculated on an Apple M2 CPU.

### Application to simulated data

As a first validation of the framework, we generated a synthetic time series and imposed an underlying time-varying connectivity pattern. More specifically, we generated a 10-dimensional Gaussian random signal with zero mean and unit variance, consisting of 5 chunks, each 1000 time-frames long. We then generated 5 random covariance matrices, and multiplied each chunk with the Cholesky Decomposition of one of these matrices. This procedure imposed a different covariance matrix on each chunk of the signal.

Following the DySCo framework we computed the sliding window covariance matrix using a window size of 121 frames, (we use odd numbers to make windows symmetric). We computed the reconfiguration speed using the 1,2 and *∞*-distances and a *τ* value of 100 frames. Distances were computed on the matrices normalised by their norms. Finally, we computed the Functional Connectivity Dynamics matrices using the distances 1,2 and Infinity.

### Application to task fMRI

#### HCP task fMRI data.

We used a sample of pre-processed task-based (working memory N-Back)(fMRI (tfMRI) data from the Human Connectome Project (HCP)(S1200) to illustrate the applicability of this framework to real data. Detailed overview of the task fMRI acquisition details can be found at ([72], www.humanconnectome.org), to summarise: whole brain EPI acquisitions were acquired with a 32 channel head-coil in a modified 3T Siemens Skyra. The images were acquired with a TR = 720 ms, TE = 33.1 ms, flip angle = 52°, BW = 2290 Hz/Px, in-plane FOV = 208 × 180 mm, 72 slices, 2.0 mm isotropic voxels, with a multi-band acceleration factor of 8 [73]. During task-based acquisitions two runs were conducted employing opposing phase encoding for each run. Minimal pre-processing is used according to [46] but several steps including: removal of spatial distortions, realignment of volumes compensating for subject motion, registration to structural data, bias field reduction, normalisation the 4D image to a global mean, and masking of the data with the final brain mask.

In each run n-back task data was collected, which includes 8 blocks of 10 trials per block. Each block commences with a 2.5s cue that describes task type e.g. 2-back or target image (0-back). Within each trial an image is presented to the participant and requires a button press per image. If the image matches the previous image (0-back, 4 blocks of the 8 block design) or was observed two images prior to the current image (2-back, 4 blocks of the 8 block design) then the participant should press a button with their right index finger. If the image is non-matching to either of these conditions then the participant should press a button with their right middle finger. There were a total of 4 distinct categories of images presented (tools, body-parts, neutral faces, and places). Each image category was presented in 2 of the blocks (for both the 0 back and 2-back) across the total 16 blocks in the two runs.

From the HCP pipeline we took each of the different task conditions as described above and generated a single task timecourse for the HCP Working Memory task (illustrated in DySCo analysis of the HCP dataset) by: (i) convolving each of the timecourses for each of the eight conditions with a double gamma canonical hemodynamic response function (HRF); (ii) subsequently, we took the mean value for each TR across all conditions resulting in a single timecourse.

We randomly sampled 100 pre-processed tfMRI participants from the available 1,034 subjects in the HCP dataset. From these we constructed functional time series data of the cortical hemispheres using python Nibabel libraries [74]. As this is simply an illustration of the potential of this framework, we decided to only use the left-hemisphere. This led to a 32492 by 405 matrix corresponding to the total voxels and TRs respectively. We then filtered this array by removing voxels containing either 0 or null values (as these correspond to tissue boundaries).

We expected our measures to temporally align with the task paradigm, confirming their dynamic sensitivity. Specifically, we expected the reconfiguration speed to present peaks corresponding to the switching between task and rest, and the FCD matrix to reflect this task-rest structure. Moreover, we expected the eigenvalue spectrum of the dynamic matrix to be different in task versus rest, which should be highlighted by the Von Neumann Entropy.

#### Preliminary exploration of the DySCo matrices.

The first step is the choice of the most suitable dFC matrix in the DySCo framework to apply to the data. We did that by both visual inspection and quantification of the similarity between different matrices. For the visual inspection, we randomly selected two signals from a subject and plotted the sliding window correlation in time, the sliding window covariance, both with a window size from 5 to 50, the co-fluctuation and the instantaneous Phase Alignment. See Results (Application of voxel-level dFC to the HCP dataset).

We expected two facts from the Theory: that the instantaneous Phase Alignment value should be similar to the sliding window correlation at a specific time-scale of observation (See Mathematical structure of the DySCo dFC matrices) and that the co-fluctuation should be similar to a sliding window covariance with minimal window size (See Mathematical structure of the DySCo dFC matrices).

For the equivalence between sliding window correlation and **iPA** at a specific time-scale, we expect that there is a specific window size that maximises the similarity between the instantaneous Phase Alignment and the sliding window correlation. We expect this peak to be dependent on the bandwidth of the signals. Therefore, we selected 500 random couples of signals for each of the subjects and for each couple of signals we computed the similarity (using Pearson Correlation) between the time trace of the instantaneous Phase Alignment and the sliding window correlation at all the different window sizes. Note that to be computed the instantaneous Phase Alignment requires the data to be bandpassed. We bandpassed the data in three frequency ranges, 0.01-0.04 Hz, 0.01-0.08 Hz, 0.05-0.1 Hz. We underline that 0.01-0.08 Hz is the gold standard frequency range adopted for fMRI data [17, 34, 37]. However, we also tried a lower and higher bandwidth to check our expectation that the bandwidth plays the same role that the window size plays in the correlation matrix.

For the equivalence between a sliding window of size 1 and co-fluctuation, we repeated the same analysis above to compare co-fluctuation and sliding window covariance with different window sizes. Please note that we did not conduct additional comparisons (for example correlation with covariance) because they represent quantities that are conceptually different.

Based on our investigation (see Application of voxel-level dFC to the HCP dataset) we decided to proceed using the sliding window correlation matrix.

#### Voxel level sliding window correlation analysis of HCP data.

Using the results from the preliminary analysis we next applied the DySCo framework to compute sliding window correlation in the whole sample of 100 participants (window size = 21, number of eigenvectors = 10). From the obtained eigenvectors and eigenvalues we calculated the following DySCo measures: Reconfiguration Speed, the Functional Connectivity Dynamics (FCD) matrix, (see Distances between dFC operators), Von Neumann Entropy. To show the sensitivity of the Von Neumann entropy and reconfiguration speed we calculated the correlation between these measures and the original task time-course using Pearson’s correlation coefficient. To assess the sensitivity of these measures at the single-subject level we repeated the analysis on a single example subject chosen from the available participants. In the results we show measures of entropy, speed, and FCD matrices for all subjects as well as at the individual subject. For visualisation purposes we also extracted the 3 eigenvectors associated with the 3 biggest eigenvalues of the dFC matrix.

## Supporting information

S1 Fig Sensitivity analysis robustness of the results to the change in window size. The figure shows the mean von Neumann Entropy, with each colour of the line denoting the specific window size for the calculation.
(TIF)

S1 Appendix Mathematical derivations analytical derivation of all the results. (PDF)
