# Supplementary figures and images for: DySCo: A general framework for dynamic functional connectivity
Source: PLoS Comput Biol. 2025 Mar 7;21(3):e1012795. doi: 10.1371/journal.pcbi.1012795 (PMC11902199; doi:10.1371/journal.pcbi.1012795)

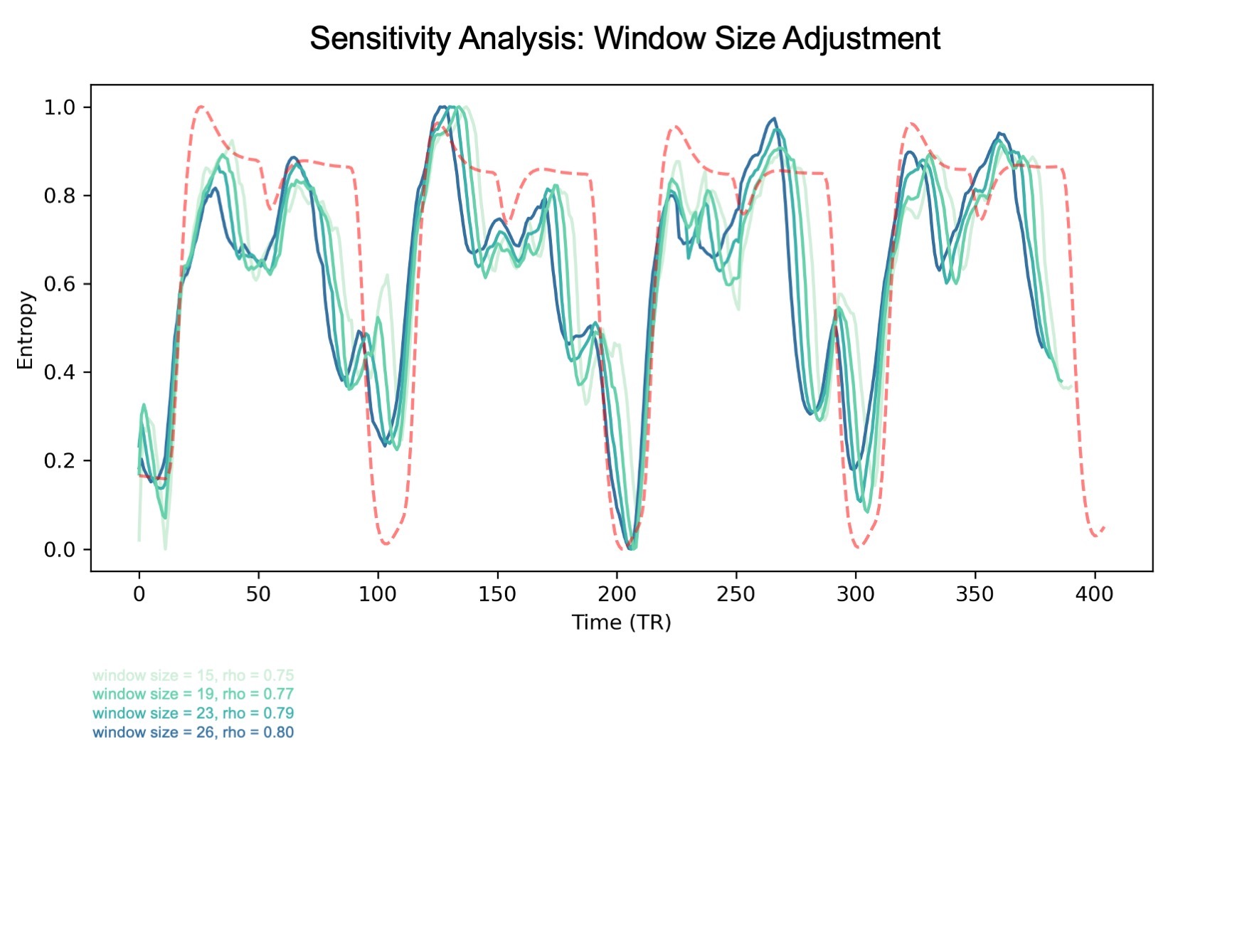

Supplement: S1 Fig — The figure shows the mean von Neumann Entropy, with each colour of the line denoting the specific window size for the calculation. (TIF) [file pcbi.1012795.s001.tif]
