# Supplementary material for: DySCo: A general framework for dynamic functional connectivity
Source: PLoS Comput Biol. 2025 Mar 7;21(3):e1012795. doi: 10.1371/journal.pcbi.1012795 (PMC11902199; doi:10.1371/journal.pcbi.1012795)
Supplement: S1 Appendix — (PDF) [file pcbi.1012795.s002.pdf]

Mathematical Derivations for *DySCo: a general framework for dynamic Functional Connectivity*

February 5, 2025

S1 Appendix

S1.1 dFC matrices

We show now in an extended form how to compute $\mathbf{C}(t)$ matrices.

S1.1.1 Sliding window covariance and correlation

Let us start from N signals $x_1(t), x_2(t), ..., x_N(t)$ which compose a vector that evolves with time $\mathbf{x}(t) \in \mathbb{R}^N$, in a window of time $t = 1...T$.

The estimator of the covariance matrix Cov of each couple of signals (i.e. the element ij of the covariance matrix) is:

$$\mathrm{Cov}_{ij} = \frac{1}{T-1}\sum_{t=1}^{T}(x_i(t) - \bar{x_i})(x_j(t) - \bar{x_j}) \tag{S1}$$

Here, $\bar{x_i}$ is the mean of the signal $x_i(t)$ in the window.

If we redefine the signals $y_i(t) = x_i(t) - \bar{x_i}$, then: $\mathrm{Cov}_{ij} = \frac{1}{T-1}\sum_{t=1}^{T} y_i(t)y_j(t)$. With a more compact notation, $\mathrm{Cov} = \frac{1}{T-1}\sum_{t=1}^{T}\mathbf{y}(t)\mathbf{y}(t)^\top$, where $\mathbf{y}(t) = (y_1(t), y_2(t), ...y_N(t))^\top$.
The same holds for a sliding window correlation matrix. Here,

$$\mathrm{Corr}_{ij} = \frac{\sum_{t=1}^{T}(x_i(t) - \bar{x_i})(x_j(t) - \bar{x_j})}{\sqrt{\sum_{t=1}^{T}(x_i(t) - \bar{x_i})^2}\sqrt{\sum_{t=1}^{T}(x_j - \bar{x_j})^2}}. \tag{S2}$$

If we z-score the signals, i.e. we define the signals $z_1(t), z_2(t)...z_N(t), t = 1...T$ as the z-score of $x_1, x_2...x_N$, in the window:

$$z_i(t) = \frac{x_i(t) - \bar{x_i}}{\sqrt{\sum_{t=1}^{T}(x_i(t) - \bar{x_i})^2}} \tag{S3}$$

Then:

$$\text{Corr} = \frac{1}{T-1}\sum_{t=1}^{T}\mathbf{z}(t)\mathbf{z}(t)^{\top} \tag{S4}$$

When the sliding window covariance or correlation matrices are computed using a weighted/tapered window, the equations above hold true, with a preliminary step of weighting the signals $\mathbf{x}(t)$ with the weights $w(t)$ within the $t = 1...T$ window of observation.

The DySCo framework also applies to the sample partial correlation. The partial correlation between two signals $x_i(t)$ and $x_j(t)$, controlling for a set of variables $\mathbf{W}$, can be expressed as the correlation between their residuals from linear regressions on $\mathbf{W}$:

$$\text{Corr}_{ij|W} = \text{corr}(e_i(t), e_j(t)),$$

where: $e_i(t)$ are the residuals obtained by regressing $x_i(t)$ based on the set of variables $\mathbf{W}$.

Thus, the partial correlation can be also inserted in the DySCo framework as long as the following equation is applied to the (z-scored) residuals $\mathbf{z_e}$ and not to the signals themselves:

$$\text{Corr}_W = \frac{1}{T-1}\sum_{t=1}^{T}\mathbf{z_e}(t)\mathbf{z_e}(t)^{\top} \tag{S5}$$

Similar considerations apply also for the sample Spearman correlation. The Spearman correlation between two signals $x_i(t)$ and $x_j(t)$ is defined as the Pearson correlation between their rank-transformed values:

$$\text{Spearman}_{ij} = \text{corr}(\text{rank}(x_i(t)), \text{rank}(x_j(t))),$$

where $\text{rank}(x_i(t))$ represents the ranks of the values of $x_i(t)$ at each time point t.

Thus, the Spearman correlation can also be incorporated into the DySCo framework by applying the following equation to the rank-transformed (z-scored) signals $\mathbf{z_r}$, rather than to the original signals:

$$\text{Spearman} = \frac{1}{T-1}\sum_{t=1}^{T}\mathbf{z_r}(t)\mathbf{z_r}(t)^{\top} \tag{S6}$$

S1.1.2 Co-fluctuation matrix

Another widely employed matrix is the co-fluctuation matrix. To compute the co-fluctuation matrix, signals are z-scored to obtain $\zeta(t)$ in the whole recording (not anymore in the sliding window of size T) . Then, the co-fluctuation matrix is:

$$\mathrm{Cof}(t) = \zeta(t)\zeta(t)^\top \tag{S7}$$

This is analogous to a sliding window covariance matrix where the window size is minimal and equal to 1.

S1.1.3 Approaches based on instantaneous phase differences

When brain areas are modelled as narrow band oscillators, it is possible to define dFC matrices based on the instantaneous phases of signals $\theta_i(t)$. This approach overcomes the limitations of selecting a window of observation of the signals, however, it introduces the need of selecting a band of interest. Indeed, the assumption of a narrowband means that a specific time t_0, we can *locally* approximate signals $x_i(t_0)$ with oscillators that have the same frequency, an instantaneous amplitude $A_i(t_0)$ and an instantaneous phase $\theta_i(t_0)$. Phases can be extracted using the Hilbert or wavelet transform. See [1] for an accurate description of this method.

It should be noted that the correlation coefficient of two pure sinusoidal waves over their common period is equal to the cosine of their phase shift:

$$\mathrm{corr}(A_i \cos(2\pi ft - \theta_i), A_j \cos(2\pi ft - \theta_j)) = \cos(\theta_i - \theta_j) \tag{S8}$$

Therefore, once the instantaneous phases of the signals $\theta_i(t)$ are computed, the **iPA** is analogously defined:

$$\mathbf{iPA}_{ij}(t) = \cos(\theta_i(t) - \theta_j(t)) \tag{S9}$$

Each entry, similarly to the case of sliding window correlation matrix, is 1 if the signals have exactly the same instantaneous phase, -1 if they are in anti-phase, 0 if the two phases are orthogonal. Similarly to the correlation matrix, the **iPA** matrix has a fixed trace of N.

Given that the matrix $\mathbf{iPA}_{ij} = \cos(\theta_i - \theta_j) = \cos(\theta_i)\cos(\theta_j) + \sin(\theta_i)\sin(\theta_j)$, the **iPA** matrix can be decomposed into a dyadic sum of two terms. We define the "cosine" vector $\mathbf{c} = (\cos(\theta_1), \cos(\theta_2), ..., \cos(\theta_N))^\top \in \mathbb{R}^N$, and the "sine" vector $\mathbf{s} = (\sin(\theta_1), \sin(\theta_2), ..., \sin(\theta_N))^\top \in \mathbb{R}^N$, and rewrite the matrix as:

$$\mathbf{iPA} = \mathbf{cc}^\top + \mathbf{ss}^\top \tag{S10}$$

As explained in Section "Mathematical structure of the DySCo dFC matrices" (main text), the other two approaches to compute dFC based on complex phasors are the following:

- **Sliding Window Phase Locking Matrix**: the Phase Locking Matrix quantifies if there is a constant phase delay of the signals in the window [2]. It is the complex counterpart of the **iPA** matrix. It also requires to extract the instantaneous phases of signals:

$$\mathbf{PL}(t) = \frac{1}{T}\sum_{\tau=t-T/2}^{\tau=t+T/2} \exp(i\theta(\tau))\exp(i\theta(\tau))^H \tag{S11}$$

where $\exp(i\theta(\tau)) = \begin{bmatrix} e^{i\theta_1(\tau)} \\ e^{i\theta_2(\tau)} \\ \vdots \\ e^{i\theta_N(\tau)} \end{bmatrix}$ is the vector of the element-wise complex exponentials of the phases of the signals and H denotes its hermitian conjugate.

- **Wavelet Coherence Spectrum**: the Complex Wavelet Coherence Matrix quantifies the coherence between signals based on their wavelet transforms [3, 4]. It requires computing the wavelet transform of the signals and their complex coefficients:

$$\mathbf{CWC_f}(t) = \mathbf{W}_f(t)\mathbf{W}_f(t)^H \tag{S12}$$

where $W_f(t) = \begin{bmatrix} \frac{w_{f,1}(t)}{||w_{f,1}(t)||} \\ \frac{w_{f,2}(t)}{||w_{f,2}(t)||} \\ \vdots \\ \frac{w_{f,N}(t)}{||w_{f,N}(t)||} \end{bmatrix}$ is the complex vector of normalized wavelet coefficients of the signals at time τ and frequency f. Similarly to the Phase Locking matrix, these values can also be averaged in a window [3, 4].

S1.2 A general result for the DySCo matrices

Equation 1 (main text) can be extended to any matrix of the type

$$C_{ij} = \sum_t f(x_i(t), x_j(t))$$

where the function f is of the form:

$$f(x_i(t), x_j(t)) = \underbrace{\varphi(x_i(t)) + \varphi(x_j(t))}_{[1]} + \underbrace{\psi(x_i(t) - x_j(t))}_{[2]} + \underbrace{\sum_k \pi_k(x_i(t))\pi_k(x_j(t))}_{[3]}$$

where φ and π_k are general functions, while ψ is an even function that is periodic or has a finite support.

S1.2.1 Formal proof

It is sufficient to show that the three separate pieces of $f(x_i, x_j)$ allow an expansion as in Equation 1 (main text). Then, the final expansion will be the sum for all t of the three separate expansions:

- **[1]** $\varphi(x_i) + \varphi(x_j)$**:** In matrix form, this is

$$1\varphi^\top + \varphi 1^\top,$$

where the φ vector is the element-wise

$$\varphi = \begin{bmatrix} \varphi(x_1) \\ \vdots \\ \varphi(x_N) \end{bmatrix},$$

and the 1 vector is

$$1 = \begin{bmatrix} 1 \\ \vdots \\ 1 \end{bmatrix}.$$

Thus, the matrix $1\varphi^\top + \varphi 1^\top$ has an analytical rank-2 EVD (for the full derivation see Section S1.2.2):

$$\lambda_{1\varphi}\mathbf{u}_{1\varphi}\mathbf{u}_{1\varphi}^\top + \lambda_{2\varphi}\mathbf{u}_{2\varphi}\mathbf{u}_{2\varphi}^\top.$$

- **[2]** $\psi(x_i - x_j)$**:** Here, ψ is an even function that is periodic or has a finite support $[a, b]$ (this is true for any practical measure on signals). Let us assume without loss of generality that $[a, b] = [-\pi, \pi]$. Thus, the function can be expanded in Fourier series as follows:

$$\psi(x_i - x_j) = \sum_h a_h \cos(hx_i - hx_j).$$

In matrix form, this is

$$\sum_h a_h(\mathbf{c}_h\, \mathbf{c}_h^\top + \mathbf{s}_h\, \mathbf{s}_h^\top),$$

where $\mathbf{c}_h$ is

$$\mathbf{c}_h = \begin{bmatrix} \cos(hx_1) \\ \vdots \\ \cos(hx_N) \end{bmatrix}.$$

, and same for $\mathbf{s}_h$ (see the derivation of the **iPA** matrix in Section S1.1.3).

- **[3]** $\sum_k \pi_k(x_i)\pi_k(x_j)$**:** This is trivially, in matrix form,

$$\sum_k \mathbf{p}_k\mathbf{p}_k^\top,$$

where the vector $\mathbf{p}_k$ is

$$\mathbf{p}_k = \begin{bmatrix} \pi_k(x_1) \\ \vdots \\ \pi_k(x_N) \end{bmatrix}.$$

By adding these terms together and summing in the window, the matrix $\mathbf{C}$ can be written following Equation 1 (main text):

$$\mathbf{C} = \sum_t \Big[\lambda_{1\varphi}(t)\mathbf{u}_{1\varphi}(t)\mathbf{u}_{1\varphi}(t)^\top + \lambda_{2\varphi}(t)\mathbf{u}_{2\varphi}(t)\mathbf{u}_{2\varphi}(t)^\top + \sum_h a_h \mathbf{c}_h(t)\,\mathbf{c}_h(t)^\top$$

$$+ \sum_h a_h \mathbf{s}_h(t)\,\mathbf{s}_h(t)^\top + \sum_k \mathbf{p}_k(t)\mathbf{p}_k(t)^\top\Big].$$

S1.2.2 Lemma: fast EVD of $1\varphi^\top + \varphi 1^\top$

Here we show that the matrix $1\varphi^\top + \varphi 1^\top$ admits an EVD by a simple decomposition of a 2×2 matrix. This is needed to prove the above result.

The eigenvectors $\mathbf{u}_{1,2}$ will be a linear combination of 1 and φ,
thus $\mathbf{u}_{1,2} = a_{1,2}1 + b_{1,2}\varphi$. Thus, by imposing the eigenvector equation:

$$\left(1\varphi^T + \varphi 1^T\right)(a1 + b\varphi) = \lambda(a1 + b\varphi)$$

Expanding:

$$1\varphi^T 1a + 1\varphi^T \varphi b + \varphi 1^T 1a + \varphi 1^T \varphi b = \lambda a1 + \lambda b\varphi$$

We define $\varphi^T 1 = 1^T\varphi = \delta$,

and

$$\varphi^T\varphi = \|\varphi\|^2, \quad 1^T 1 = N$$

By assuming linear independence between 1 and φ in the general case:

$$\begin{cases} \delta a + \|\varphi\|^2 b = \lambda a \\ Na + \delta b = \lambda b \end{cases}$$

This is a 2x2 eigenvector equation for the coefficients a and b:

$$\begin{bmatrix} \delta & \|\varphi\|^2 \\ N & \delta \end{bmatrix} \begin{bmatrix} a \\ b \end{bmatrix} = \lambda \begin{bmatrix} a \\ b \end{bmatrix}$$

which has the following solution: the eigenvalues are:

$$\lambda_{1,2} = \delta \pm \sqrt{N}\|\varphi\|$$

The eigenvector components are:

$$\begin{bmatrix} a \\ b \end{bmatrix} = \begin{bmatrix} \pm\frac{\|\varphi\|}{\sqrt{N}} \\ 1 \end{bmatrix}$$

Thus:

$$\mathbf{u}_1 = \frac{\|\varphi\|}{\sqrt{N}}1 + \varphi$$

$$\mathbf{u}_2 = -\frac{\|\varphi\|}{\sqrt{N}}1 + \varphi$$

S1.3 Eigendecomposition of a dyadic sum

S1.3.1 SVD of the data matrix and Temporal Covariance EVD

Here we relate the eigenvalue decomposition of a dyadic sum $\mathbf{C} = \sum_{t=1}^{T} \mathbf{x}_t \mathbf{x}_t^\top = \mathbf{X}\mathbf{X}^\top \in \mathbb{R}^{N\times N}$ to the singular value decomposition (SVD) of the data matrix $\mathbf{X} \in \mathbb{R}^{N\times T}$, and consequently to the eigenvalue decomposition of the Temporal Covariance matrix $\mathbf{R} = \mathbf{X}^\top\mathbf{X} \in \mathbb{R}^{T\times T}$.

The data matrix can be expressed as its SVD decomposition $\mathbf{X} = \mathbf{U}\mathbf{\Sigma}\mathbf{V}^\top$, where $\mathbf{U}$ and $\mathbf{V}$ are respectively $N \times N$ and $T \times T$ orthogonal matrices, and $\mathbf{\Sigma}$ is an $N \times T$ rectangular diagonal matrix with non-negative entries, the singular values, $\sigma_{ii} = \mathbf{\Sigma}_{ii}$ [5]. The columns u_i of $\mathbf{U}$ and the columns v_i of $\mathbf{V}$ are called the left-singular vectors the right-singular vectors of $\mathbf{X}$, respectively.

From $\mathbf{C} = \mathbf{X}\mathbf{X}^\top = \mathbf{U}\mathbf{\Sigma}\mathbf{\Sigma}^\top\mathbf{U}^\top$ and $\mathbf{R} = \mathbf{X}^\top\mathbf{X} = \mathbf{V}\mathbf{\Sigma}^\top\mathbf{\Sigma}\mathbf{V}^\top$, it is straightforward to observe that:

- The matrices $\mathbf{C}$ or $\mathbf{R}$ share the same non-null eigenvalues, the non-null diagonal entries of $\mathbf{\Sigma}^\top\mathbf{\Sigma}$ or, equivalently, $\mathbf{\Sigma}\mathbf{\Sigma}^\top$, which are the squared singular values of $\mathbf{X}$.
- The eigenvectors of the connectivity matrix, columns of $\mathbf{U}$, and the eigenvectors of the Temporal Covariance matrix, columns of $\mathbf{V}$, are the left-singular vectors and the right-singular vectors of $\mathbf{X}$, respectively. In particular, the following relation holds $\mathbf{U}\mathbf{\Sigma} = \mathbf{X}\mathbf{V}$.

S1.3.2 General weighted sum

Here we provide a general proof of the formula for the eigenvector decomposition of a matrix $\mathbf{C}$ expressed as a weighted dyadic sum:

$$\mathbf{C} = \sum_{t=1}^{T} w_t \mathbf{x}_t \mathbf{x}_t^\top \tag{S13}$$

This formula considers both positive and negative weights w_t.

This matrix is a linear operator that outputs any input vector in the space spanned by the T vectors $\mathbf{x}_t$. Therefore, it has rank no greater than T. Its first T

eigenvectors $\mathbf{u}_i$ belong as well to the space spanned by the $\mathbf{x}_t$ vectors. Therefore, they will be a linear combination of the $\mathbf{x}_t$ vectors: for an eigenvector $\mathbf{u}_i$, it must be $\mathbf{u}_i = \sum_{j=1}^{T} v_{ji}\mathbf{x}_j$. Therefore, the problem of finding the eigenvectors of $\mathbf{C}$ is equivalent to finding v_{ji}.

By imposing the eigenvector-eigenvalue equation, i.e. $\mathbf{C}\mathbf{u}_i = \lambda_i \mathbf{u}_i$, and imposing that $\mathbf{u}_i$ is a linear combination of $\mathbf{x}_t$, we can write:

$$\sum_{t=1}^{T} w_t \mathbf{x}_t \mathbf{x}_t^\top \sum_{j=1}^{T} v_{ji}\mathbf{x}_j = \lambda_i \sum_{j=1}^{T} v_{ji}\mathbf{x}_j \tag{S14}$$

By rearranging terms we can write:

$$\sum_{t=1}^{T}\sum_{j=1}^{T} w_t v_{ji} \mathbf{x}_t^\top \mathbf{x}_j \mathbf{x}_t = \lambda_i \sum_{t=1}^{T} v_{it}\mathbf{x}_t \tag{S15}$$

By imposing that $\mathbf{x}_t$ are linearly independent, we have that:

$$w_t \sum_{u=1}^{T} \mathbf{x}_t^\top \mathbf{x}_j v_{ji} = \lambda_i v_{it} \;\; \forall t \tag{S16}$$

If we define the vector $\mathbf{v}_i = (v_{1i}, v_{2i}, ...v_{Ti})^\top$, and the matrix $\mathbf{R}_{tj} = w_t \mathbf{x}_t^\top \mathbf{x}_j$ equation S16 can be rewritten in matrix notation as:

$$\mathbf{R}\,\mathbf{v}_i = \lambda_i \mathbf{v}_i \tag{S17}$$

.

This is an eigenequation for the Temporal Covariance matrix. The eigenvalues of the Temporal Covariance matrix $\mathbf{R}$ will therefore be the eigenvalues of the connectivity matrix $\mathbf{C}$. The eigenvectors of the connectivity matrix $\mathbf{u}_i$ will be related to the eigenvectors of the Temporal Covariance matrix $\mathbf{v}_i$ by formula $\mathbf{u}_i = \sum_{j=1}^{T} v_{ji}\mathbf{x}_j$.

S1.4 Computation of distance of dFC matrices in the DySCo framework

Finally, we show here that the distances in the DySCo framework can all be computed from the eigenvector representation of the dFC matrices, without the need of rebuilding the matrices and explicitly computing the norm of their difference.

Having the dyadic representations $\mathbf{C}(t_1) = \sum_{i=1}^{T} \lambda_i(t_1)\mathbf{u}_i(t_1)\mathbf{u}_i(t_1)^\top$ and $\mathbf{C}(t_2) = \sum_{i=1}^{T} \lambda_i(t_2)\mathbf{u}_i(t_2)\mathbf{u}_i(t_2)^\top$ available, we can combine them to express the difference matrix $\mathbf{C}(t_1) - \mathbf{C}(t_2) = \sum_{j=1}^{2T} w_j \mathbf{d}_j \mathbf{d}_j^\top$, where $w_j = \lambda_i(t_1)$ and $\mathbf{d}_j = \mathbf{u}_j(t_1)$ for $j \leq T$ and $w_j = -\lambda_{j-T}(t_2)$ and $\mathbf{d}_j = \mathbf{u}_{j-T}(t_2)$ for $j > T$. Therefore, it is possible to resort to the TCEVD in S1.3 to compute the eigenvalues of $\mathbf{C}(t_1) - \mathbf{C}(t_2)$, using a Temporal Covariance matrix of size $2T$. Once the eigenvalues are computed, it is possible to compute the norm of $\mathbf{C}(t_1) - \mathbf{C}(t_2)$, i.e. the distance.

S1.4.1 Frobenius case

In case of the distance 2, it is possible to compute the distance in an even faster fashion which does not require the eigendecomposition of the Temporal Covariance matrix.

Indeed, since $\mathbf{C}(t_1) - \mathbf{C}(t_2)$ is symmetric, it holds:

$$\begin{aligned} \| \mathbf{C}(t_1) - \mathbf{C}(t_2) \|_2 = \mathrm{Tr}(\mathbf{C}(t_1) - \mathbf{C}(t_2))^2 = \\ \| \mathbf{C}(t_1) \|_2 + \| \mathbf{C}(t_2) \|_2 - 2\mathrm{Tr}(\mathbf{C}(t_1)^\top \mathbf{C}(t_2)) = \\ \| \mathbf{C}(t_1) \|_2 + \| \mathbf{C}(t_2) \|_2 - 2\sum_{ij} \mathbf{C}(t_1)_{ij} \mathbf{C}(t_2)_{ij} \end{aligned} \tag{S18}$$

The separated norms of $\mathbf{C}(t_1)$ and $\mathbf{C}(t_2)$ are already provided by knowledge of their eigenvalues. We note also that $\mathrm{Tr}(\mathbf{C}(t_1)^\top \mathbf{C}(t_2))$ is the Frobenius product between the two matrices, which may be a quantity of interest to compute on itself as explained in the main text (Section "Distances between dFC operators").

The fast way to compute the Frobenius product, which does not require to expand the matrices, is the following:

$\mathrm{Tr}(\mathbf{C}(t_1)^\top \mathbf{C}(t_2)) = \mathrm{Tr}(V(t_1)D(t_1)V(t_1)^\top V(t_2)D(t_2)V(t_2)^\top)$ by eigenvector decomposition $= \mathrm{Tr}(V(t_1)D(t_1)^{1/2}D(t_1)^{1/2}V(t_1)^\top V(t_2)D(t_2)^{1/2}D(t_2)^{1/2}V(t_2)^\top) =$ $\mathrm{Tr}(W(t_1)W(t_1)^\top W(t_2)W(t_2)^\top)$ by redefinition of $W(t) = V(t)D(t)^{1/2}$. For the circular property of the trace $\mathrm{Tr}(W(t_1)W(t_1)^\top W(t_2)W(t_2)^\top) =$ $\mathrm{Tr}(W(t_1)^\top W(t_2)W(t_2)^\top W(t_1)) = \| W(t_1)^\top W(t_2) \|_2$, where $W(t_1)^\top W(t_2)$ is a $T \times T$ small matrix, and its 2-norm can be computed by just summing all its squared elements.

S1.4.2 Alignment

Finally, we show that the Frobenius distance between the projector matrices of $\mathbf{C}(t_1)$ and $\mathbf{C}(t_2)$ on a subset of their eigenvectors is a measure of alignment of these eigenvectors. Let us define $U_n(t)$ as the submatrix of the first n columns of U. The projector matrix on these n eigenvectors of $\mathbf{C}(t)$ is $U_n(t)U_n(t)^\top$. Thus, the Frobenius distance between the projector matrices of $\mathbf{C}(t_1)$ and $\mathbf{C}(t_2)$ is:

$$\begin{aligned} \| U_n(t_1)U_n(t_1)^\top - U_n(t_2)U_n(t_2)^\top \|_2 = \\ \| U_n(t_1)U_n(t_1)^\top \|_2 + \| U_n(t_2)U_n(t_2)^\top \|_2 - \\ 2\mathrm{Tr}(U_n(t_1)U_n(t_1)^\top U_n(t_2)U_n(t_2)^\top) = \\ 2(n - \| U_n^\top(t_1)U_n(t_2) \|_2) \end{aligned} \tag{S19}$$

This means that this distance depends on the 2-norm of $U_n^\top(t_1)U_n(t_2)$, which is a matrix of all the scalar products between the eigenvectors of $\mathbf{C}(t_1)$ and $\mathbf{C}(t_2)$. In case the eigenvectors match perfectly, the 2-norm of $U_n^\top(t_1)U_n(t_2)$ is maximal and equal to n, which makes the distance go to zero. On the opposite side, when

the eigenvectors are all completely orthogonal the norm of $U_n^\top(t_1)U_n(t_2)$ goes to zero making the distance maximal and equal to $2n$.

S1.5 On the estimation of covariance/correlation matrices

A formal problem in dFC is the estimation of high-dimensional matrices from a few data points. This problem is inherited from FC analysis that typically suffers from the same limitation, for example when considering low-time resolution data such as fMRI: with a parcellation of $N = 100$ regions, there are 2500 entries to be estimated from a few time points, typically a few hundreds.

One explanation why FC can still provide valuable insights is the effective dimensionality of the data being low enough: only a small number of eigenvalues are significantly different from 0. This allows to estimate the covariance of a large number of channels using a small number of time points.

DySCo shows that, provided that we consider similarity measures, it is always possible to see dFC matrices as a matrix $XX^\top$, where X is the data matrix. Thus, the eigenvectors are the right singular vectors of the X matrix, where X has been preprocessed based on what measure is being computed, e.g. for correlation signals are z-scored, for phase locking we are taking the cosine of the angle, etc.

Thus, performing an SVD of X is always formally valid, even when the number of samples is small. The SVD will still be measuring the geometry of the signals, i.e. the shape of the cloud in Fig. 1, and the DySCo measures quantify how this cloud changes in time. When the window size converges to 1, i.e. in the case of the co-fluctuation matrix, the eigenvector converges to the signal itself. We make it clear that, in this case, we are studying the sample statistic, which may end up being the population statistic or not, based on the specific application.

The question remains, of course, whether any real information is captured by the geometry or it is only noise. The correlation between experimental designs and metrics derived from DySCo analysis, or any other kind of analysis, serves as a validation step.

References

[1] Giuseppe de Alteriis, Eilidh MacNicol, Fran Hancock, Alessandro Ciaramella, Diana Cash, Paul Expert, and Federico E Turkheimer. Eida: A lossless approach for dynamic functional connectivity; application to fmri data of a model of ageing. *Imaging Neuroscience*, 2:1–22, 2024.

[2] Sergul Aydore, Dimitrios Pantazis, and Richard M Leahy. A note on the phase locking value and its properties. *Neuroimage*, 74:231–244, 2013.

[3] Aslak Grinsted, John C Moore, and Svetlana Jevrejeva. Application of the cross wavelet transform and wavelet coherence to geophysical time series. *Nonlinear processes in geophysics*, 11(5/6):561–566, 2004.

[4] Mario Chavez and Bernard Cazelles. Detecting dynamic spatial correlation patterns with generalized wavelet coherence and non-stationary surrogate data. *Scientific reports*, 9(1):7389, 2019.

[5] Gilbert Strang. *Linear algebra and its applications*. 2012.
